# Factors associated with stimulant persistence among children with ADHD: a retrospective cohort study

**DOI:** 10.3389/fphar.2026.1847068

**Published:** 2026-07-10

**Authors:** Tehila Fisher Yosef, Tamar Landau, Ran Levy, Zohar Shalev, Gabriel Chodick, Tal Patalon, Arik Eisenkraft

**Affiliations:** 1 Maccabi Healthcare Services, Health Division, Tel Aviv, Israel; 2 Gray Faculty of Medical and Health Sciences, Tel-Aviv University, Tel-Aviv, Israel; 3 Arison School of Business, Reichman University, Herzliya, Israel; 4 Kahn Sagol Maccabi Research and Innovation Center, Maccabi Healthcare Services, Tel-Aviv, Israel; 5 The Institute for Research in Military Medicine, The Hebrew University Faculty of Medicine, Jerusalem, Israel

**Keywords:** ADHD, adherence, pediatrics, pharmacoepidemiology, stimulants

## Abstract

**Importance:**

Optimizing ADHD stimulant therapy in children is difficult due to individual variability and lack of reliable response predictors, forcing a trial-and-error approach that burdens both patients and families.

**Objective:**

To identify factors associated with treatment success based on real-world patterns of ADHD stimulant use.

**Design:**

Setting and participants: In this retrospective cohort study we used the comprehensive electronic health records of Maccabi Healthcare Services. We included children aged 6–18 years diagnosed with ADHD between 2015–2023 who had purchased at least one ADHD stimulant and had at least 1 year of follow-up. Patients were classified as persistent users once they purchased at least eight prescriptions over a fixed 12-month period, with each purchase covering a 30-day period, and there were no gaps of 90 days or more; others were defined as *non-persistent*. Data was stratified by stimulant type using the WHO Anatomical Therapeutic Chemical classification. Demographic and clinical covariates were analyzed to identify factors associated with sustained medication use. The study followed the STROBE Statement.

**Results:**

The cohort comprised of 43,825 children with ADHD, of which 18,783 (43%) were Persistent users. Persistent users were more often of male sex (67.5% vs. 55.4%), lower socioeconomic status (44.7% vs. 42.4% with low or very low SES), and initiated treatment at a younger age (76.4% vs. 52.9% initiated treatment at 6–12 years). Lisdexamfetamine was associated with the highest odds of treatment persistence when used as the initial stimulant, compared with the reference group (OR = 2.2, CI = 1.86–2.59), although this finding was based on a relatively small sample (n = 654).

**Conclusion and Relevance:**

In this retrospective cohort study of pediatric patients with ADHD, male sex, low socioeconomic status, early treatment initiation, and first-line lisdexamfetamine were associated with higher persistence.

## Introduction

In the last decades, there is an increase in the prevalence of attention-deficit/hyperactivity disorder (ADHD) diagnosis both in the US (Chung et al., 2019; Xu et al., 2018; Abdelnour et al., 2022) and in Israel (Davidovitch et al., 2017). Consequently, there is an increase in pharmacological treatment of ADHD (Danciu, 2011) and stimulant medications have become the first line of pharmacological treatment in pediatric ADHD (Advokat et al., 2013). Optimizing drug therapy for ADHD is challenging due to substantial variability in treatment response and tolerability responses across patients (Hermens et al., 2005; Swanson et al., 2011; Ogrim et al., 2014; Hawk et al., 2018; Ogrim and Kropotov, 2019).

Currently, no reliable predictors guide optimal stimulant selection, often necessitating a trial-and-error approach that may burden patients and families; therefore, adherence may be hard to achieve. (Hermens et al., 2005; Ogrim and Kropotov, 2019; Cedergren et al., 2022; Alavi et al., 2020). Several systematic reviews examining medication adherence among children and adolescents with ADHD reported wide variability across studies (Ferrin et al., 2025; Karimi et al., 2025) and concluded that medication adherence and persistence are generally poor and have shown little improvement over time.

In this pharmaco-epidemiological study, we aimed to identify predictors of treatment persistence in pediatric ADHD. This population differs substantially from adults, as diagnosis, treatment decisions, medication adherence, and long-term outcomes are strongly influenced by ongoing neurodevelopment, family involvement, school functioning, and caregiver preferences (Cortese et al., 2018). In addition, ADHD treatment is commonly initiated during childhood (Peterson et al., 2024), making this population especially relevant for evaluating long-term medication patterns. This study may support clinical decision-making and improve treatment outcomes in pediatric ADHD.

## Materials and methods

### Setting and study design

The study was conducted using the databases of Maccabi Healthcare Services (MHS), Israel. MHS, the second largest state-mandated healthcare provider, serves over 2.5 million members and has maintained comprehensive electronic medical records since the early 1990s. The study was approved by the Maccabi Ethics Committee (Approval number MHS-0091-23, from 12 October 2023), and all collected data was de-identified, and informed consent was waived. The study was funded by a grant from the Research Institute of Maccabi Healthcare Services–the Kahn-Sagol Maccabi (KSM) Institute. The study followed the STROBE (Strengthening the Reporting of Observational Studies in Epidemiology) Statement (Cuschieri, 2019).

### Study population

In Israel, ADHD may be diagnosed as early as preschool age, from approximately 4 years of age. However, ADHD stimulants are not approved or routinely prescribed below the age of six (Davidovitch et al., 2017). Therefore, the use of stimulants in preschool-aged children is possible but is considered off-label and remains relatively uncommon in Israel.

The study therefore included 1) all children aged 6–18 years who were diagnosed with ADHD (ICD-9 codes 314.0, 314.00, 314.01, 314.9) by a neurologist and/or psychiatrist and/or an ADHD board certified specialist and/or primary physicians between the years 2009–2023; 2) had purchased at least one type of medication for this medical condition; and 3) had at least 1 year follow-up in MHS records after medication purchase. The time frame of 2009–2023 was chosen since the first time the Israeli Ministry of Health has issued official guidelines for the diagnosis of ADHD was in April 2008 (Director General Circular No 8/2008 IM of H, 2008). These included clear definitions on who is allowed to diagnose the condition. Follow-up was retrospectively defined from the index date (first stimulant purchase) and continued until the earliest of the following: end of the study period (31 December 2023), reaching 18 years of age, or disenrollment from the Maccabi Healthcare Services registry for any reason, including death.

**FIGURE 1:**
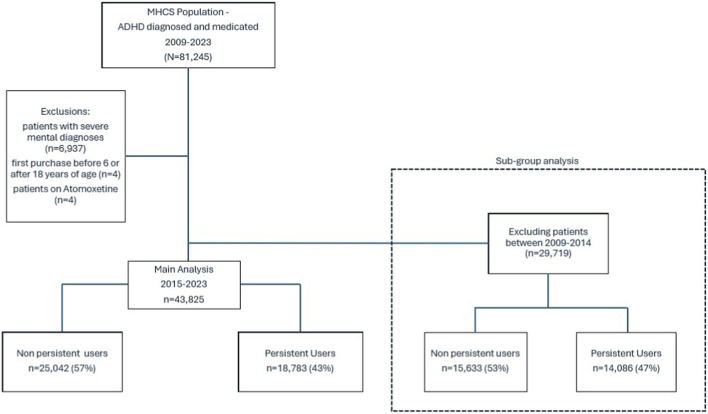
Flow chart of the study design. Patients from 2009 to 2014 were excluded from the main analysis as only methylphenidate was available. However, we performed a sub-group analysis of this group, as depicted in the figure. MHCS–Maccabi Healthcare Services.

The study group was defined as “Persistent Users”, and it included patients which purchased at least 8 monthly prescriptions from the same stimulant class over a rolling 1 year period, with each purchase covering a 30-day period, and there were no gaps of 90 days or more. In previous studies (Ferrin et al., 2025; Wehmeier et al., 2015; Ibrahim, 2002) adherence was measured by proportion of days covered (PDC) or by medication possession ratios (MPR) and the follow up time varied from several months to a calendar year. However, these criteria are not reliable when applied to the Israeli population: even though it is not the recommended practice, in Israel treatment is usually provided exclusively during school days which are approximately only 8 months cumulatively in a calendar year (Cohen et al., 2016). Based on observed seasonal variation in medication purchases, calendar months were categorized to reflect school and extended vacation periods (April, July, and August). Calendar year was included in the multivariable logistic regression model to assess a potential interaction with persistence. The rest of the patients were included in the control group (“Non-Persistent Users”). Figure 1 describes the study design. Both groups were divided into three categories by age: 6–12-year-olds (representing elementary school age), 13–16-year-olds (representing Israeli middle schoolers), and 17–18-year-olds (representing Israeli senior highschoolers). We have also employed several methods of stratification of the data, based on age, sex, and medication groups according to the World Health Organization’s Anatomical Therapeutic Chemical (ATC) classification system (available at https://atcddd.fhi.no/atc_ddd_index/, accessed on 8 September 2025).

Exclusion criteria were defined as children with a history of mental disorders (see detailed list of diagnoses in Supplementary Table 1). Individuals with co-morbid mental disorders may experience greater symptom severity and distinct patterns of medication use and adherence (Faber et al., 2010; Sikirica et al., 2014). Therefore, this population was considered clinically distinct and was excluded from the analysis.

### Data collection and statistical analysis

Our study is a comparative analysis of treatment initiation and is specifically designed to address the question: Among newly diagnosed children with ADHD, which initial stimulant leads to better adherence and persistence?’.

The main metric outcome was persistent purchasing of a specific stimulant, as defined by Anatomical Therapeutic Chemical (ATC) 5, considered as a measure of treatment adherence. These included methylphenidate (N06BA04), dexmethylphenidate (N06BA11), methamphetamine (N06BA03), dexamphetamine (N06BA02), and lisdexamfetamine (N06BA12). Atomoxetine (N06BA09) was excluded as it was prescribed to only four patients; therefore, the analysis focused specifically on stimulant medications rather than on ADHD medications as a whole.

Independent variables included patients’ demographics at the time of the first prescription (e.g., age, gender, household residential socioeconomic status) and parental psychiatric history (ICD-9 diagnosis in Supplementary Table 2). Residential socioeconomic status (SES) was based on a score built by Points Location Intelligence for commercial purposes using geographic information system (GIS). The score is calculated at the enumeration-area level (approximately zip code size) using data on retail expenditures, employment, car ownership, and housing. A detailed description of the collected variables is available in Table 1 and Supplementary Table 3. Baseline characteristics were summarized using descriptive statistics. Continuous variables were presented as medians with interquartile ranges (IQR) and compared between groups using the Wilcoxon rank-sum test. Categorical variables were reported as counts and percentages, with differences evaluated using the Chi-square test. Standardized mean differences (SMDs) were calculated to assess the magnitude of between-group imbalances.

**TABLE 1:** Characteristics of the study population by persistent use of ADHD therapy.

Characteristics	Non-persistent users (n = 25,042; 57%) n (%)	Persistent users (n = 18,783; 43%) n (%)	SMD**
Sex, no. (%)			0.25
Male	13,873 (55.4)	12,670 (67.5)	
Female	11,169 (44.6)	6,113 (32.5)	
Age at ADHD diagnosis (median [IQR])	10.47 [8.2,13.8]	8.58 [7.2,11. 1]	0.52
Age at first ADHD Stimulant (median [IQR])	11.62 [9.1,14.6]	9.10 [7.6,11.8]	0.64
Age at first at ADHD stimulant no. (%), y			0.53
6-12	13,259 (52.9)	14,344 (76.4)	
13-16	8,314 (33.2)	3,628 (19.3)	
17-18	3,469 (13.9)	811 (4.3)	
Time from ADHD diagnosis to first medication (median [IQR]), y	0.08 [0,0.8]	0.04 [0,0.4]	0.23
Follow up (years) (median [IQR])*, y	2 [1,4]	5 [3,7]	0.97
Follow-up time without anti-ADHD therapy (mean (SD)), y	0.46 (1.05)	0.26 (0.72)	0.22
Calendar year of index date (median [IQR])	2019 [2017–2021]	2018 [2016–2020]	0.27
Parental psychiatric diagnosis, no. (%)	9,312 (37.2)	7,242 (38.6)	0.03
SES, no. (%)			0.08
High	5,536 (22.1)	4,179 (22.2)	
Medium	8,891 (35.5)	6,217 (33.1)	
Low	8,457 (33.8)	6,378 (34)	
Very low	2,158 (8.6)	2,009 (10.7)	
First ADHD stimulant, no. (%)			0.12
Dexmethylphenidate	416 (1.7)	390 (2.1)	
Dextroamphetamine	3042 (12.1)	1657 (8.8)	
Lisdexamfetamine	314 (1.3)	340 (1.8)	
Methylphenidate	21,270 (84.9)	16,396 (87.3)	
No. of medication groups (median [IQR])	1 [1,2]	2 [1,2]	0.45

* Sensitivity analysis for the follow-up time is provided in Supplementary Tables 4, 5.

** All baseline characteristics differed significantly between groups (p < 0.001), except for parental psychiatric history, for which no statistically significant difference was observed. Abbreviations: ADHD, attention deficit disorder; SES, Socioeconomic status. Sex–Male/Female. SMD, standardized mean differences.

Candidate variables were selected *a priori* based on their clinical relevance and availability within the dataset and included demographic and medication-related characteristics. Variables associated with treatment persistence in univariable analyses were entered into the multivariable logistic regression model (Table 2).

**TABLE 2:** Multivariable-adjusted odds ratios (OR) and 95% confidence intervals for ADHD.

Variable		Adj. OR^a^	(95% CI)	P-value
Sex	Males vs. females	1.47	(1.41–1.53)	<0.001
SES	Very low	1 (ref.)	​	​
	Low	0.83	(0.78–0.90)	<0.001
	Medium	0.81	(0.76–0.87)	<0.001
	High	0.96	(0.89–1.04)	0.29
Age group	17–18	1 (ref.)	​	​
	6–12	4.29	(3.93–4.69)	<0.001
	13–16	1.67	(1.52–1.84)	<0.001
Index calendar year (per year)		0.77	(0.75–0.78)	<0.001
Time from ADHD diagnosis to stimulant (per 1 year)		0.80	(0.79–0.81)	<0.001
First ADHD stimulant	Methylphenidate	1 (ref.)	​	​
	Dextroamphetamine	1.01	(0.94–1.08)	0.76
	Dexmethylphenidate	1.38	(1.19–1.60)	<0.001
	Lisdexamfetamine	2.2	(1.86–2.59)	<0.001

^a^ The logistic regression model included all variables in the table.

SES, socioeconomic status; OR, odds ratio; CI, confidence intervals; ADHD, attention deficient hyperactivity disorder, ref reference.

To address potential follow-up time bias, we conducted sensitivity analyses with exact matching on follow-up time. To assess association between patient characteristics, medication-related factors and persistent use, we applied multivariable logistic regression model, reporting odds ratios (ORs) with 95% confidence intervals (CIs) and p-values. Heatmaps were generated to visualize consistency patterns across age groups and first medication types. Stratified plots were used to illustrate differences of age, sex and medication use in consistency trends.

During the preliminary data analysis, we found that between the years 2009 and 2014, only methylphenidate was approved and available in MHS for pediatric ADHD. All other medications were introduced, approved and equally available in MHS only after 2014. Therefore, the comparative analysis focused on the years 2015–2023. A subgroup analysis was conducted to further explore demographic and clinical differences between persistent and non-persistent users for patients with purchases between 2009 and 2014, when only methylphenidate was available.

All analyses were performed using *R version 4.4.1*, with two-sided p-values <0.05 considered statistically significant.

## Results

**FIGURE 2:**
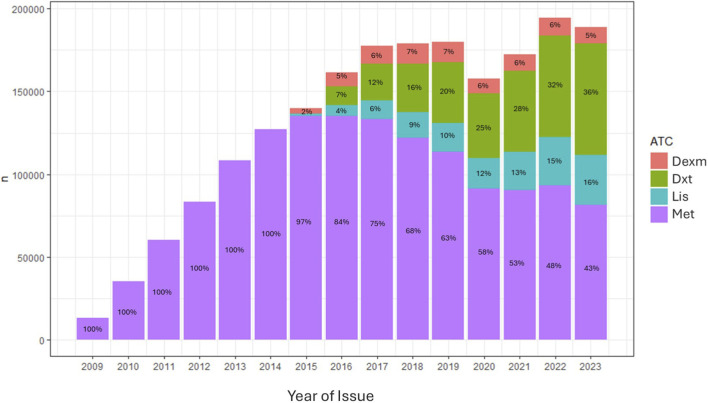
Total purchases of four different ADHD stimulants (Dexmethylphenidate in red, Dextroamphetamine in green, Lisdexamfetamine in blue, and Methylphenidate in purple), as defined in the Anatomical Therapeutic Chemical (ATC) group list, from 2009 to 2023. The y-axis represents the number of purchases, while the x-axis shows the years. This graph effectively illustrates the changing landscape of ADHD stimulants prescriptions over a 15-year period, highlighting shifts in prescription patterns and the introduction of new medications. In each column we present the relative percentage of each drug group purchased during the year.

A total of 81,245 children with a diagnosis of ADHD and with at least one purchase of stimulants within MHS were identified during 2009–2023. Of them, 6,937 children had a diagnosis of another mental disorder, and were excluded (Figure 1). As mentioned earlier, during the years 2009–2014 only methylphenidate was available in Israel, therefore this group was extracted from the main analysis (n = 29,719). As only 4 patients were treated with Atomoxetine, and only 4 patients started treatment before the age of 6 or after the age of 18, these groups were also excluded from final analysis (Figure 1). Figure 2 depicts the annual ADHD stimulant purchases in the study group, including the data prior to 2015. After exclusion was completed, the study population included a total of 43,825 children (Table 1; Figure 1). Of these, 18,783 (43%) were classified as “persistent users,” while 25,042 (57%) were classified as “non-persistent users”.

Baseline characteristics stratified by initial stimulant group are presented in Supplementary Table 3. Differences were observed across stimulant groups with respect to age, sex, calendar year of treatment initiation, and follow-up duration. These variables were considered in multivariable analyses.

We found significant differences between the “non-persistent” and “persistent” groups across the various characteristics (Table 1). Persistent users were more likely to be of male sex (67.5% vs. 55.4%) and reside in very low SES areas (10.7% vs. 8.6%). Methylphenidate was the most common first-line stimulant in both groups, but more prevalent in the “persistent” group (87.3% vs. 84.9%).

Parental psychiatric history was evaluated according to ICD-9 diagnosis (Supplementary Table 2) from the parent’s medical records. The analysis was restricted to parents who were insured in the same HMO as their child. Results show a high parental psychiatric history rate at both groups (37%–38%) and no significant difference.

Persistent group had lower use of dextroamphetamine as first stimulant (8.8% vs. 12.1%, p < 0.001, SMD = 0.119) and a higher median number of stimulant group attempts (2 vs. 1, p < 0.001, SMD = 0.445). This group was also younger at first diagnosis (8.6 vs. 10.5 years) and medication initiation (9.1 vs. 11.6 years), with moderate to large effect sizes (SMD = 0.516 and 0.640, both p < 0.001). Stimulant initiation was more common at ages 6–12 in the persistent group (76.4% vs. 52.9%), whereas the non-persistent group initiated treatment at an older age (p < 0.001, SMD = 0.526).

Multivariate logistic regression was performed to evaluate associations between persistent medication use and potential predictors. (Table 2). Male sex was associated with 47% higher odds of medication persistence (OR 1.47, 95% CI 1.41–1.53, p < 0.001). Compared to the reference group (very low SES), all other SES levels, except the high-SES group, showed significantly lower odds for persistent use. Compared with adolescents (17–18), preschool children (6–12) had substantially higher odds of treatment persistence (OR = 4.29, 95% CI 3.93–4.69), see more details in the Supplemental data analysis.

Calendar index year was significantly associated with persistent use, with earlier calendar years demonstrating higher persistence rates (Table 2). To evaluate whether the association between treatment (stimulant groups) and persistence varied over time, we tested an interaction term between treatment and calendar year. The interaction term was not statistically significant, and inclusion of this term did not materially alter the treatment effect estimates. In addition, an exploratory analysis of medication purchases by calendar month demonstrated lower purchase rates during extended school breaks (April, July, and August), as shown in Supplementary Figure 1.

**FIGURE 3:**
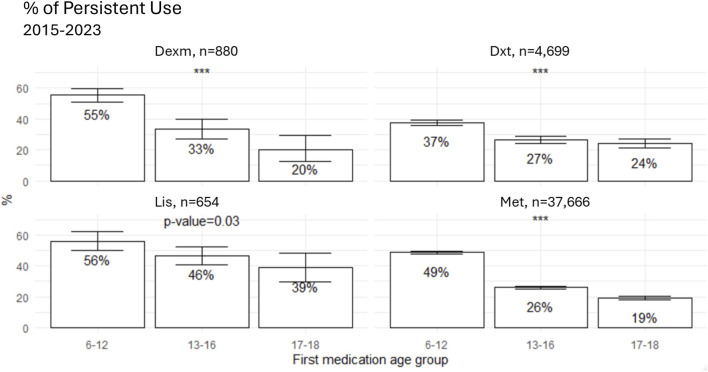
Percent of persistent users during 2015–2023 by first medication and age group, with 95% CI. The figure displays the four medication subclasses (Dextroamphetamine, Methylphenidate, Dexmethylphenidate, and Lisdexamfetamine). Across all medication subclasses, persistent users were more likely to initiate medication at younger ages (notably 6–12 years). This pattern is less evident for the Lisdexamfetamine groups, where users exhibit similar persistent rate across all age groups (p-value = 0.03).

Across all treatment groups, younger children demonstrated both higher rates of stimulant initiation and greater persistence over time (Figure 3). Moreover, the proportion of persistent users decreased with age, while the proportion of non-persistent users increased. This pattern was also observed when analyses was stratified by sex. This observation was also evident in the years 2009–2014, when only methylphenidate was available. Although this trend was observed with all stimulants, this pattern was less pronounced in the lisdexamfetamine group, where users demonstrated a relatively persistent rate across all age groups, with a notably higher rate observed at older ages compared to the other groups, and substantial proportion of persistent users were still present among those who initiated treatment during middle school age (Figure 3).

First line therapy was significantly associated with treatment persistence (p < 0.001), with lisdexamfetamine showing the highest persistent use (Supplementary Figure 3). Stratification by age showed higher persistence rate in the younger age group, with lisdexamfetamine demonstrating a more uniform persistence across all age groups (Supplementary Figure 4).

Variation in index year and follow-up duration revealed potential follow-up time bias (median follow-up 2 vs. 5 years), which was addressed in a sensitivity analysis. The results remained consistent with the primary analysis, including the direction of the association between medication type and treatment persistence. Although the magnitude of the association for lisdexamfetamine was attenuated, it remained statistically significant (OR = 1.79, 95% CI 1.47–2.19) (Supplementary Tables 4, 5). Though longer follow-up would be expected to bias estimates toward higher event probability in the study group, the persistence of similar effect sizes after restricting follow-up suggests robustness of the findings.

We also conducted a subgroup analysis of children exposed to a single medication, including those with persistent use from the first treatment attempt and those who discontinued pharmacological treatment after the initial medication.

Persistence differed significantly by medication: methylphenidate predominated in younger children, while lisdexamfetamine showed nearly twice the persistence rate in middle school–aged children.

During the years 2009–2014 when only methylphenidate was available to treat ADHD in MHS, we found significant demographic and clinical differences, reflecting a similar pattern to that found in the primary analysis (Supplementary Table 6). Persistent users were more likely to be of the male sex (65.6% vs. 58.9%, SMD = 0.14, p < 0.001) similar the results in the main analysis. However, differences in SES distribution in the subgroup analysis differed from those observed in the main analysis, with the study group showing a higher proportion of participants in the high SES category (25.8% vs. 22.3%, SMD = 0.104, p < 0.001) and fewer in the low SES category (30.8% vs. 34.6%). Persistent users were diagnosed and initiated treatment at a younger age, with mean ages at first diagnosis and first medication of 9.75 and 9.87 years, respectively, compared to 11.56 and 11.74 years among non-persistent users (SMD >0.56 for both, p < 0.001). Age group distributions at treatment initiation also differed markedly: 75.9% of persistent users started treatment in the primary school age group (6–12), compared to 53.1% in the non-persistent group, while older age groups were more represented among non-persistent users (SMD = 0.495, p < 0.001). We also stratified the persistent users by sex and age (Supplementary Figures 5a–d) and found similar results in both primary and sub-group analyses.

## Discussion

Our results indicate that early initiation of stimulants for children with ADHD, particularly during primary school years, is associated with a higher likelihood of achieving persistent use of the same medication. These findings corroborate with those of international studies (W et al., 2025; Efron et al., 2020; Huang et al., 2021). A possible explanation for this phenomenon is that children diagnosed at a younger age tend to present with a more pronounced externalizing manifestation of ADHD (Brancati et al., 2023), making them more likely to require pharmacological treatment and adhere to it. In addition, during childhood, treatment decisions are primarily made by parents, whereas during adolescence, complying with treatment decisions is more challenging in any therapeutic context (Barnard-Brak et al., 2023; Schein et al., 2022).

Patients classified as non-persistent also experienced a substantially longer delay between diagnosis and the initiation of pharmacological therapy. This pattern may reflect lower perceived need for treatment, greater ambivalence toward pharmacotherapy, or milder ADHD symptoms. In contrast, persistent users initiated treatment quicker and at a younger age, which may indicate more pronounced symptoms at baseline, necessitating consistent daily pharmacological treatment and limiting the feasibility of dose omission. The shorter the interval between diagnosis and treatment initiation, the greater the likelihood of treatment persistence, which is intuitive as it likely reflects higher clinical need and stronger motivation for pharmacological treatment.

Unlike other ADHD stimulants, which showed lower adherence rates when initiated during adolescence, lisdexamfetamine was associated with relatively higher persistence rate. This agrees with the results of a robust double-blind, placebo-controlled trial where adolescents prescribed with lisdexamfetamine showed high rates of treatment responses, with success rates measured as symptom improvement and persistence comparable to or slightly exceeding those of methylphenidate (Newcorn et al., 2017). One possible explanation for its success in this age group is its proposed mechanism of action, which focuses more on impulse control (Andrada Santos and Estrin, 2024) a highly relevant factor during adolescence (Nunn et al., 2020).

Given the non-randomized treatment allocation and the relatively small number of lisdexamfetamine users, observed differences in baseline characteristics between stimulant groups may reflect real-world prescribing practices, temporal changes in medication uptake, and underlying differences in patient populations. The observed differences may influence treatment selection and persistence patterns, including indication, side-effect profiles, dosing convenience, clinician preference, and family characteristics. Lisdexamfetamine’s long-acting formulation may be particularly suited to adolescents with extended school days and may be less likely to be discontinued. In contrast, methylphenidate is available in multiple formulations with varying durations of action, which we could not distinguish, potentially introducing bias. These limitations underscore the need for randomized studies to better assess differences between stimulants. It should be noted that, in contrast to other health management organizations in Israel, throughout the entire study period lisdexamfetamine was freely available within MHS and could be prescribed without prior failure of other medications or progression through previous lines of treatment. Therefore, no treatment-line bias or third-line treatment effect is present in this study. While these limitations should be considered when interpreting the findings, the observed association between lisdexamfetamine initiation and greater treatment persistence remained consistent across the primary and sensitivity analyses.

Additional predictors of successful persistent use included male sex and lower socioeconomic status. Multiple studies report that during elementary school years male sex is associated with a higher likelihood of both being prescribed a stimulant for ADHD and achieving persistence and adherence (Morgan and Hu, 2023). A possible explanation of this phenomenon is the difference in symptoms between boys and girls with boys more likely than girls to exhibit externalizing symptoms (Skogli et al., 2013; Waddell and McCarthy, 2010), which are more disruptive and thus more frequently recognized or referred for evaluation. As a result, boys are more likely to be diagnosed and are more frequently referred for pharmacological treatment by professionals (Kle et al., 2021) – with a greater likelihood of remaining on treatment.

Interestingly, these sex differences were less evident in the older age groups of our study (13 years and above). This corroborated with previous studies that found that among clinically diagnosed children with ADHD, the severity of symptoms was similar between boys and girls as they entered adolescence, despite the earlier disparity favoring higher identification among boys during elementary years (Mowlem et al., 2019; De Ronda et al., 2024).

As demonstrated in this study, studies from the UK observe a “social gradient” in ADHD stimulant prescribing and persistence; Children with ADHD from lower socioeconomic background are more likely to be prescribed stimulants (Nunn et al., 2020) possibly due to higher prevalence, greater perceived need, or more severe symptoms recognized in these groups. Additionally, it is possible that lower socioeconomic groups lack the financial and time resources required for complementary interventions, and as a result, are more likely to rely on pharmacological treatment and adhere to it more persistently.

Overall, the number of ADHD stimulant purchases among the study population increased over the years, with one notable exception: a slight decline observed in 2020 (COVID year). The increase in the number of prescriptions for ADHD in our study corresponds with the rising prevalence of the disorder in the pediatric population in Israel over the years (Davidovitch et al., 2017; Shehadeh-Sh et al., 2023).

This may partly reflect changing parental attitudes, with diagnosis and medication increasingly viewed as tools to support academic performance (Ragadran et al., 2023).

The similar results in both primary and sub-group analyses of persistent users when stratified by sex and age, indicate that the higher proportion of persistent use among younger children of male sex is driven primarily by overall ADHD treatment patterns rather than by the specific type of stimulant (Supplementary Figures 5a–d).

### Clinical implications

The findings of our study suggest that while in the elementary school age group there are several options with approximately 50% chance of reaching adherence (lisdexamfetamine, methylphenidate, Dexmethylphenidate), during adolescence there’s a significantly greater chance of reaching adherence with lisdexamfetamine as a first line stimulant. Lisdexamfetamine had highest odds for treatment persistence across all age groups as the first line stimulant. As this observation is based on a relatively small number of first line lisdexamfetamine users, further controlled studies are needed to validate this finding as a potential marker.

In our study, failure to achieve persistent use with lisdexamfetamine and dextroamphetamine predicted additional failures to achieve therapeutic adherence with other medications as well.

### Strengths

The strength of this study lies in its large study population and the high-quality data, which included comprehensive demographic information.

### Limitations

A limitation of the study is the fact it was conducted on Israeli population only, which may exhibit unique patterns of medication use that are not necessarily generalizable to other populations worldwide.

While treatment recommendations for ADHD are often extrapolated across countries, it is important to recognize that many aspects of ADHD management in children are not based on large double-blind randomized controlled trials but rather on expert consensus and accumulated clinical experience (Coghill et al., 2023). Therefore, large-scale real-world epidemiological studies from a single country can provide valuable insights that may inform clinical practice and treatment decisions in other healthcare settings as well.

The exclusion of patients with psychiatric comorbidities, including anxiety disorders, may limit the generalizability of the findings. As anxiety disorders are common among children and adolescents with ADHD (Njardvik et al., 2025), the study population may not fully reflect the broader clinical population treated in routine practice.

Another limitation is that while medication adherence is an important process outcome and may correlate with clinical response in ADHD, it should not be used as a standalone surrogate for treatment success. Discontinuation may reflect milder cases managed non-pharmacologically, while persistence does not necessarily indicate full functional recovery, symptom improvement, or treatment effectiveness. Such data, if available, exists only in unstructured form and was beyond the scope of this study. As a result, the present findings should be interpreted as reflecting patterns of continued medication use rather than clinical outcomes.

Several potential confounders were beyond the scope of this study, including concomitant medications and comorbidities other than mental disorders.

A formal wash-out period was not applied, and treatment initiation was defined according to the first recorded ADHD stimulant purchase within the database. As a result, prior stimulant use occurring before the available observation period could not be identified, potentially leading to misclassification of some patients as new users, although the potential impact is likely limited given the study population was predominantly composed of children aged 6–12 years and stimulant treatment in Israel is generally initiated from age 6 onward.

The MHS database does not include information on parental education level, academic functioning, or quality of life, which may also act as potential confounders. However, a previous study (Shehadeh-Sh et al., 2023) among Israeli parents of children with ADHD showed that although parental education is a strong predictor of seeking a medical diagnosis, it is not associated with medication adherenceand is therefore unlikely to bias our results.

Another limitation of this study lies with the constraints of the ATC system; our data included information at the level of the active pharmaceutical ingredient but did not allow differentiation between immediate-release and extended-release formulations. Finally, the study included only medicated children with ADHD and did not examine the broader ADHD population or non-pharmacological interventions.

## Conclusion

In this retrospective cohort study of pediatric patients with ADHD, male sex, low socioeconomic status, early treatment initiation and first line lisdexamfetamine were associated with treatment persistence.

These findings should be interpreted with caution, and additional studies are needed to evaluate the impact of treatment selection, switching patterns, and other clinical factors on long-term medication use.

## Data Availability

The datasets presented in this article are not readily available because according to the information security policy of Maccabi Healthcare Services, the anonymized data is accessible only to researchers approved by the ethics committee and is not available to the general public upon request.
