# Supplementary material for: Factors associated with stimulant persistence among children with ADHD: a retrospective cohort study
Source: Front Pharmacol. 2026 Jul 10;17:1847068. doi: 10.3389/fphar.2026.1847068 (PMC13395766; doi:10.3389/fphar.2026.1847068)
Supplement: Supplementary file 1 [file DataSheet1.pdf]

Supplemental Appendix

Table of Contents

Supplemental Tables

Supplemental Table 1. Mental disorder diagnoses excluded from the study........ 2
Supplemental Table 2. Parental psychiatric history included in the analysis........ 9
Supplemental Table 3. Demographic characterizations of the patients stratified by first stimulant purchased 22
Supplemental Table 4. Multivariate Logistic regression for Sensitivity analysis using a generalized linear model (GLM) with exact matching on follow-up time 24

Supplemental Table 5. Demographic characterizations of patients included in the sensitivity analyses with exact matching on follow-up time........ 25

Supplemental Table 6. Demographic characterizations of the patients included in the Sub-Analysis of years 2009-2014 when only methylphenidate was available to treat ADHD in Israel. 26

Supplemental Figures

Supplemental Figure 1. Monthly distribution of ADHD medication purchases across the study period 27
Supplemental Figure 2. Proportion of patients who used only one medication group throughout the study period, stratified by age group and medication class 28
Supplemental Figure 3. The association between the first medication purchased and the medication persistently used by the patient........ 29

Supplemental Figure 4. Heatmaps showing consistency of medication use by first medication type and age group........ 30
Supplemental Figure 5a-d. Percent of persistent users by age and sex, with 95% CI........ 32

Supplemental Tables

Supplemental Table 1. Mental disorder diagnoses excluded from the study

icd 9 code	Diagnosis Group	Diagnosis
296.4	BIPOLAR	BIPOLAR DISORDER MOST RECENT EPISODE MANIC
296.40	BIPOLAR	BIPOLAR DIS MOST RECENT EPIS HYPOMAN
296.40	BIPOLAR	BIPOLAR DIS SINGLE MANIC EPIS
296.44	BIPOLAR	BIPOLAR DISORDER MOST RECENT EPISODE MANIC SEVERE + PSYCHOTIC FEATURES
296.44	BIPOLAR	BIPOLAR DIS SINGLE MANIC EPISODE SEVERE + PSYCHOTIC FEATURES
296.46	BIPOLAR	BIPOLAR DIS MOST RECENT EPIS MANIA IN FULL REMISSION
296.46	BIPOLAR	BIPOLAR DIS SINGLE MAN EPIS FULL REMISSION
296.5	BIPOLAR	BIPOLAR DIS MOST RECENT EPIS DEPRESSED
296.5	BIPOLAR	BIPOLAR AFFECTIVE DISORDER-DEPRESSED
296.51	BIPOLAR	BIPOLAR DIS MOST RECENT EPIS MILD
296.52	BIPOLAR	BIPOLAR DIS MOST RECENT EPISODE MODERATE
296.54	BIPOLAR	BIPOLAR DIS MOST RECENT EPIS SEVERE + PSYCHOTIC FEATURES
296.56	BIPOLAR	BIPOLAR DIS MOST RECENT EPIS FULL REMISSION
296.56	BIPOLAR	BIPOLAR AFFECTIVE DIS DEPRESSED IN REMISSION
296.6	BIPOLAR	BIPOLAR AFFECTIVE DISORDER-MIXED
296.60	BIPOLAR	BIPOLAR DIS MOST RECENT EPIS MIX
296.64	BIPOLAR	BIPOLAR DIS MOST RECENT EPIS MIXED SEVERE + PSYCHOTIC FEATURES
296.65	BIPOLAR	BIPOLAR AFFECTIVE DISORDER MIXED PART REMISSION
296.66	BIPOLAR	BIPOLAR DIS MOST RECENT EPIS MIX FULL REMISSION
296.66	BIPOLAR	BIPOLAR AFFECTIVE DISORDER MIXED IN REMISSION
296.7	BIPOLAR	BIPOLAR AFFECTIVE DISORDER-UNSPECIFIED
296.80	BIPOLAR	BIPOLAR DIS NOS
296.89	BIPOLAR	BIPOLAR II DIS
296.0	MANIC DISORDER	HYPOMANIA- NOS
296.0	MANIC DISORDER	MANIC AFF. DIS.+PSYCHOTIC FEATURES

296.10	MANIC DISORDER	MANIC DISORDER-RECURRENT EPISODE
296.10	MANIC DISORDER	MANIC AFFEC. DIS RECUR SEVER+PSYCH.FEATURES
296.10	MANIC DISORDER	MANIC AFFECTIVE DIS RECURR IN FULL REMISSION
296.8	MANIC DISORDER	ATYPICAL MANIC DISORDER
296.8	MANIC DISORDER	MANIC DEPRESSION
309.0	DEPRESSION	ADJUSTMENT DISORDER ASSOCIATED WITH DEPRESSED MOOD
309.0	DEPRESSION	ADJUSTMENT DISORDER-MIXED ANXIETY AND DEPRESSIVE
309.3	COGNITIVE IMPAIRMENT	ADJUSTMENT REACTION+PREDOMINANT DISTURBANCE OF CONDUCT
309.4	COGNITIVE IMPAIRMENT	ADJUSTMENT REACTION + MIXED EMOTIONS AND CONDUCT DISTURBANCE
312.0	COGNITIVE IMPAIRMENT	UNDERSOCIALIZED CONDUCT DISORDER-AGGRESSIVE TYPE
312.1	COGNITIVE IMPAIRMENT	UNDERSOCIALIZED CONDUCT DISORDER-UNAGGRESSIVE TYPE
312.4	COGNITIVE IMPAIRMENT	MIXED DISTURBANCE OF CONDUCT AND EMOTIONS
312.8	COGNITIVE IMPAIRMENT	OTHER SPECIFIED DISTURBANCES OF CONDUCT NEC
312.9	COGNITIVE IMPAIRMENT	UNSPECIFIED DISTURBANCE OF CONDUCT
295	SCHIZOPHRENIC DISORDERS	SCHIZOPHRENIC DISORDERS
295.0	SCHIZOPHRENIC DISORDERS	SIMPLE TYPE SCHIZOPHRENIA
295.0	SCHIZOPHRENIC DISORDERS	SIMPLE TYPE SCHIZOPHRENIA-UNSPECIFIED STATE
295.01	SCHIZOPHRENIC DISORDERS	SIMPLE TYPE SCHIZOPHRENIA SUBCHRONIC STATE
295.02	SCHIZOPHRENIC DISORDERS	SIMPLE TYPE SCHIZOPHRENIA CHRONIC STATE
295.03	SCHIZOPHRENIC DISORDERS	SIMPLE TYPE SCHIZOPHRENIA SUBCHRONIC STATE WITH ACUTE EXACERBATION
295.04	SCHIZOPHRENIC DISORDERS	SIMPLE TYPE SCHIZOPHRENIA CHRONIC STATE WITH ACUTE EXACERBATION
295.05	SCHIZOPHRENIC DISORDERS	SIMPLE TYPE SCHIZOPHRENIA IN REMISSION
295.1	SCHIZOPHRENIC DISORDERS	DISORGANIZED TYPE SCHIZOPHRENIA
295.10	SCHIZOPHRENIC DISORDERS	SCHIZOPHRENIA DISORGANIZED TYPE UNS

295.11	SCHIZOPHRENIC DISORDERS	SCHIZOPHRENIA DISORGANIZED SUBCHRONIC STATE
295.12	SCHIZOPHRENIC DISORDERS	SCHIZOPHRENIA DISORGANIZED CHRONIC STATE
295.13	SCHIZOPHRENIC DISORDERS	SCHIZOPHRENIA DISORGANIZED - SUBCHRONIC+ACUTE EXACERBATION
295.14	SCHIZOPHRENIC DISORDERS	SCHIZOPHRENIA DISORGANIZED -CHR. STATE + ACUTE EXACERBATION
295.15	SCHIZOPHRENIC DISORDERS	SCHIZOPHRENIA DISORGANIZED - IN REMISSION
295.2	SCHIZOPHRENIC DISORDERS	CATATONIC TYPE SCHIZOPHRENIA
295.20	SCHIZOPHRENIC DISORDERS	SCHIZOPHRENIA CATATONIC TYPE UNS
295.21	SCHIZOPHRENIC DISORDERS	SCHIZOPHRENIA CATATONIC SUBCHRONIC STATE
295.22	SCHIZOPHRENIC DISORDERS	SCHIZOPHRENIA CATATONIC CHRONIC STATE
295.23	SCHIZOPHRENIC DISORDERS	SCHIZOPHRENIA CATATONIC SUBCHRONIC WITH ACUTE EXACERBATION
295.24	SCHIZOPHRENIC DISORDERS	SCHIZOPHRENIA CATATONIC CHR. STATE WITH ACUTE EXACERBATION
295.25	SCHIZOPHRENIC DISORDERS	SCHIZOPHRENIA CATATONIC IN REMISSION
295.3	SCHIZOPHRENIC DISORDERS	PARANOID TYPE SCHIZOPHRENIA
295.30	SCHIZOPHRENIC DISORDERS	SCHIZOPHRENIA PARANOID UNS
295.31	SCHIZOPHRENIC DISORDERS	SCHIZOPHRENIA PARANOID SUBCHRONIC STATE
295.32	SCHIZOPHRENIC DISORDERS	SCHIZOPHRENIA PARANOID CHRONIC STATE
295.33	SCHIZOPHRENIC DISORDERS	SCHIZOPHRENIA PARANOID SUBCHRONIC STATE + ACUTE EXACERBATION
295.34	SCHIZOPHRENIC DISORDERS	SCHIZOPHRENIA PARANOID CHRONIC STATE WITH ACUTE EXACERBATION

295.35	SCHIZOPHRENIC DISORDERS	SCHIZOPHRENIA PARANOID IN REMISSION
295.40	SCHIZOPHRENIC DISORDERS	SCHIZOPHRENIFORM DIS
295.40	SCHIZOPHRENIC DISORDERS	ACUTE SCHIZOPHRENIC EPISODE
295.5	SCHIZOPHRENIC DISORDERS	LATENT SCHIZOPHRENIA
295.50	SCHIZOPHRENIC DISORDERS	LATENT SCHIZOPHRENIA- UNSPECIFIED STATE
295.51	SCHIZOPHRENIC DISORDERS	LATENT SCHIZOPHRENIA- SUBCHRONIC STATE
295.52	SCHIZOPHRENIC DISORDERS	LATENT SCHIZOPHRENIA-CHRONIC STATE
295.53	SCHIZOPHRENIC DISORDERS	LATENT SCHIZOPHRENIA SUBCHRONIC+ACUTE EXACERB
295.54	SCHIZOPHRENIC DISORDERS	LATENT SCHIZOPHRENIA CHRONIC+ACUTE EXACERBATI
295.55	SCHIZOPHRENIC DISORDERS	LATENT SCHIZOPHRENIA-IN REMISSION
295.6	SCHIZOPHRENIC DISORDERS	RESIDUAL SCHIZOPHRENIA
295.60	SCHIZOPHRENIC DISORDERS	SCHIZOPHRENIA RESIDUAL UNS
295.61	SCHIZOPHRENIC DISORDERS	RESIDUAL SCHIZOPHRENIA- SUBCHRONIC STATE
295.62	SCHIZOPHRENIC DISORDERS	RESIDUAL SCHIZOPHRENIA-CHRONIC STATE
295.63	SCHIZOPHRENIC DISORDERS	RESIDUAL SCHIZOPHRENIA SUBCHRONIC+ACUTE EXACE
295.64	SCHIZOPHRENIC DISORDERS	RESIDUAL SCHIZOPHRENIA CHRONIC+ACUTE EXACERBA
295.65	SCHIZOPHRENIC DISORDERS	RESIDUAL SCHIZOPHRENIA-IN REMISSION
295.70	SCHIZOPHRENIC DISORDERS	SCHIZOAFFECTIVE DIS

295.70	SCHIZOPHRENIC DISORDERS	SCHIZO-AFFECTIVE TYPE SCHIZOPHRENIA- UNSPECIF
295.71	SCHIZOPHRENIC DISORDERS	SCHIZO-AFFECTIVE TYPE SCHIZOPHREN SUBCHRONIC
295.72	SCHIZOPHRENIC DISORDERS	SCHIZO-AFFECTIVE TYPE SCHIZOPHRENIA-CHRONIC
295.73	SCHIZOPHRENIC DISORDERS	SCHIZO-AFFECTIVE SCHIZOPHRENIA SUBCHR+ACUTE EXACERBATION
295.74	SCHIZOPHRENIC DISORDERS	SCHIZO-AFFECTIVE SCHIZOPHRENIA CHRONIC+AC EXA
295.75	SCHIZOPHRENIC DISORDERS	SCHIZO-AFFECTIVE SCHIZOPHRENIA IN REMISSION
295.8	SCHIZOPHRENIC DISORDERS	OTHER SPECIFIED TYPES OF SCHIZOPHRENIA
295.80	SCHIZOPHRENIC DISORDERS	OTHER SPECIFIED TYPES SCHIZOPHRENIA-UNSPECIFIED STATE
295.81	SCHIZOPHRENIC DISORDERS	OTHER SPECIFIED TYPES SCHIZOPHRENIA SUBCHRONIC
295.82	SCHIZOPHRENIC DISORDERS	OTHER SPECIFIED TYPES SCHIZOPHRENIA CHRONIC
295.83	SCHIZOPHRENIC DISORDERS	OTHER SPECIFIED TYPES SCHIZOP SUBCHR+ACUTE EX
295.84	SCHIZOPHRENIC DISORDERS	OTHER SPECIFIED SCHIZOPH CHRONIC+ACUTE EXACER
295.85	SCHIZOPHRENIC DISORDERS	OTHER SPECIFIED TYPES SCHIZOPHRENIA IN REMISSION
295.9	SCHIZOPHRENIC DISORDERS	UNSPECIFIED SCHIZOPHRENIA
295.90	SCHIZOPHRENIC DISORDERS	SCHIZOPHRENIA UNDIFFERENTIATED UNS
295.90	SCHIZOPHRENIC DISORDERS	UNSPECIFIED TYPE SCHIZOPHRENIA-UNSPEC STATE
295.91	SCHIZOPHRENIC DISORDERS	SCHIZOPHRENIA UNDIFFERENTIATED SUBCHRONIC STATE
295.91	SCHIZOPHRENIC DISORDERS	UNSPECIFIED TYPE SCHIZOPHRENIA SUBCHRONIC

295.92	SCHIZOPHRENIC DISORDERS	SCHIZOPHRENIA UNDIFFERENTIATED CHRONIC STATE
295.92	SCHIZOPHRENIC DISORDERS	UNSPECIFIED TYPE SCHIZOPHRENIA-CHRONIC STATE
295.93	SCHIZOPHRENIC DISORDERS	SCHIZOPHRENIA UNDIFFERENTIATED SUBCHRONIC+ACUTE EXACERBATION
295.93	SCHIZOPHRENIC DISORDERS	UNSPECIFIED TYPE SCHIZOPH SUBCHR+ACUTE EXACER
295.94	SCHIZOPHRENIC DISORDERS	SCHIZOPHRENIA UNDIFFERENTIATED-CHRONIC+ ACUTE EXACERBATION
295.94	SCHIZOPHRENIC DISORDERS	UNSPECIFIED TYPE SCHIZOPH CHRONIC+ACUTE EXACE
295.95	SCHIZOPHRENIC DISORDERS	SCHIZOPHRENIA UNDIFFERENTIATED IN REMISSION
295.95	SCHIZOPHRENIC DISORDERS	UNSPECIFIED TYPE SCHIZOPHRENIA-IN REMISSION
301.20	SCHIZOPHRENIC DISORDERS	SCHIZOID PERSONALITY DIS
301.22	SCHIZOPHRENIC DISORDERS	SCHIZOTYPAL PERSONALITY DIS
297	PARANOIA	PARANOID STATE
297.8	PARANOIA	OTHER SPECIFIED PARANOID STATES
297.9	PARANOIA	UNSPECIFIED PARANOID STATE
298.3	PARANOIA	ACUTE PARANOID REACTION
298.4	PARANOIA	PSYCHOGENIC PARANOID PSYCHOSIS
301.00	PARANOIA	PARANOID PERSONALITY DIS
296	PSYCHOSIS	AFFECTIVE PSYCHOSES
296.90	PSYCHOSIS	AFFECTIVE PSYCHOSIS UNSPECIFIED
298	PSYCHOSIS	OTHER NONORGANIC PSYCHOSES
298.1	PSYCHOSIS	EXCITATIVE TYPE PSYCHOSIS
298.8	PSYCHOSIS	OTHER AND UNSPECIFIED REACTIVE PSYCHOSIS
298.9	PSYCHOSIS	PSYCHOSIS UNSPECIFIED
299.0	PSYCHOSIS	INFANTILE PSYCHOSIS
299.1	PSYCHOSIS	DISINTEGRATIVE PSYCHOSIS
299.10	PSYCHOSIS	DISINTEGRATIVE PSYCHOSIS-CURRENT OR ACTIVE

299.11	PSYCHOSIS	DISINTEGRATIVE PSYCHOSIS-RESIDUAL STATE	
299.81	PSYCHOSIS	EARLY CHILDHOOD PSYCHOSES RESIDUAL SPECIFIED	
299.9	PSYCHOSIS	UNSPECIFIED CHILDHOOD PSYCHOSIS	
301.50	PERSONALITY DIS	HISTRIONIC PERSONALITY DIS	
301.6	PERSONALITY DIS	DEPENDENT PERSONALITY DIS	
301.7	PERSONALITY DIS	ANTISOCIAL PERSONALITY DIS	
301.81	PERSONALITY DIS	NARCISSISTIC PERSONALITY DIS	
301.82	PERSONALITY DIS	AVOIDANT PERSONALITY DIS	
301.83	PERSONALITY DIS	BORDERLINE PERSONALITY DIS	
301.9	PERSONALITY DIS	PERSONALITY DISORDER NOS	
301.9	PERSONALITY DIS	UNSPECIFIED PERSONALITY DISORDER	
V71.02	PERSONALITY DIS	CHILDHOOD OR ADOL ANTISOCIAL BEHAVIOR	?
300.01	PERSONALITY DIS	PANIC DISORDER NO AGORAPHOBIA	
300.2	PERSONALITY DIS	PHOBIC DISORDERS WITH PANIC ATTACKS	
300.21	PERSONALITY DIS	PANIC DIS+AGORAPHOBIA	
292.89	ANXIETY	HALLUCINOGEN INDUCED ANXIETY DIS	
292.89	ANXIETY	INHALANT INDUCED ANXIETY DIS	
292.89	ANXIETY	SEDATIVE HYPNO ANXIO INDUCED ANXIETY DIS	
292.89	ANXIETY	CAFFEINE INDUCED ANXIETY DIS	
292.89	ANXIETY	AMPH INDUC ANXIETY DIS	
292.89	ANXIETY	CANNABIS INDUCED ANXIETY DIS	
292.89	ANXIETY	COCAINE INDUCED ANXIETY DIS	
292.89	ANXIETY	PHENCYCLIDINE INDUCED ANXIETY DIS	
292.89	ANXIETY	OTHER/UNKNOWN SUB INDUC ANXIETY DIS	
300.00	ANXIETY	OTHER ANXIETY STATES	
300.02	ANXIETY	GENERALIZED ANXIETY DIS	
300.09	ANXIETY AND DEPRESSIVE	ANXIETY AND DEPRESSIVE REACTION	
300.4	ANXIETY AND DEPRESSIVE	ANXIETY AND DEPRESSION	
300.4	ANXIETY AND DEPRESSIVE	MIXED ANXIETY AND DEPRESSION	
308.0	ANXIETY AND DEPRESSIVE	ANXIETY REACTION	
309.1	ANXIETY AND DEPRESSIVE	MENTAL REACTION TO DISEAS(ANXIETY-DEPRESSION-ANGER-HOSTILITY	
296.2	DEPRESSION	MAJOR DEPRESSIVE DISORDER-SINGLE EPISODE	

296.24	DEPRESSION	MAJOR DEPRESSIVE DISORDER SINGLE EPISODE + PSYCHOTIC FEATURES
296.26	DEPRESSION	MAJOR DEPRESSIVE DIS SINGLE EPIS IN REMISSION
296.30	DEPRESSION	MAJOR DEPRESSIVE DISORDER RECURRENT EPISODE
296.34	DEPRESSION	MAJOR DEPRESSIVE DISORDER RECURRENT + PSYCHOTIC FEATURES
296.35	DEPRESSION	MAJOR DEPRESS AFFECT DIS RECUR PART REMISSION
296.36	DEPRESSION	MAJOR DEPRESS AFFECT DIS RECURR IN FULL REMISSION
296.82	DEPRESSION	ATYPICAL DEPRESSIVE DISORDER
311	DEPRESSION	DEPRESSIVE DISORDER NOS
317.00	COGNITIVE IMPAIRMENT	MILD MENTAL RETARDATION IQ 50-70
318	COGNITIVE IMPAIRMENT	OTHER SPECIFIED MENTAL RETARDATION
318.1	COGNITIVE IMPAIRMENT	SEVERE MENTAL RETARDATION IQ 20-34
318.2	COGNITIVE IMPAIRMENT	PROFOUND MENTAL RETARDATION IQ UNDER 20
319.9	COGNITIVE IMPAIRMENT	MENTAL RETARDATION UNSPECIFIED
V40.00	COGNITIVE IMPAIRMENT	BORDERLINE INTELLECTUAL FUNCTIONING
294.9	COGNITIVE IMPAIRMENT	COGNITIVE DISORDER NOS
310.1	COGNITIVE IMPAIRMENT	MILD COGNITIVE IMPAIRMENT

Supplemental Table 2. Parental psychiatric history included in the analysis

**ICD-9**	**Diagnosis Description**
291	Alcoholic Psychoses
291	Alcohol Withdrawal Delirium Alcoholic delirium; Delirium tremens
291.1	Alcohol Amnestic Syndrome Alcoholic polyneuritic psychosis; Korsakoff's psychosis, alcoholic; Wernicke
291.3	Alcohol Withdrawal Hallucinosis Alcoholic: hallucinosis (acute), psychosis with hallucinosis
291.4	Idiosyncratic Alcohol Intoxication Pathologic: alcohol intoxication, drunkenness
291.8	Cther Specified Alcoholic Psychosis
291.81	Alcohol Withdrawal Alcohol: abstinence syndrome or symptoms, withdrawal syndrome or symptoms

291.9	Unspecified Alcoholic Psychosis Alcoholic: mania NOS, psychosis NOS; Alcoholism (chronic) with psychosis
292.89	Other Specified DRUG ABUSE Drug
294.9	Unspecified Organic Brain Syndrome (chronic) Organic psychosis (chronic)
295	Schizophrenic Disorders
295	Simple Type Schizophrenia Schizophrenia simplex
295.01	Simple Type Schizophrenia, Subchronic State Schizophrenia simplex
295.02	Simple Type Schizophrenia, Chronic State Schizophrenia simplex
295.03	Simple Type Schizophrenia, Subchronic State With Acute Exacerbation Schizophrenia simplex
295.04	Simple Type Schizophrenia, Chronic State With Acute Exacerbation Schizophrenia simplex
295.05	Simple Type Schizophrenia, In Remission Schizophrenia simplex
295.1	Disorganized Type Schizophrenia Hebephrenia; Hebephrenic type schizophrenia
295.1	Disorganized Type Schizophrenia, Unspecified State Hebephrenia; Hebephrenic type schizophrenia
295.11	Disorganized Type Schizophrenia, Subchronic State Hebephrenia; Hebephrenic type schizophrenia
295.12	Disorganized Type Schizophrenia, Chronic State Hebephrenia; Hebephrenic type schizophrenia
295.13	Disorganized Type Schizophrenia, Subchronic State With Acute Exacerbation Hebephrenia; Hebephrenic type schizophrenia
295.14	Disorganized Type Schizophrenia, Chronic State With Acute Exacerbation Hebephrenia; Hebephrenic type schizophrenia
295.15	Disorganized Type Schizophrenia, In Remission Hebephrenia; Hebephrenic type schizophrenia
295.2	Catatonic Type Schizophrenia Catatonic (schizophrenia): agitation, excitation, excited type, stupor, withdrawn type; Schizophrenic: cataleps
295.2	Catatonic Type Schizophrenia, Unspecified State Catatonic (schizophrenia): agitation, excitation, excited type, stupor, withdrawn type; Schi
295.21	Catatonic Type Schizophrenia, Subchronic State Catatonic (schizophrenia): agitation, excitation, excited type, stupor, withdrawn type; Schi
295.22	Catatonic Type Schizophrenia, Chronic State Catatonic (schizophrenia): agitation, excitation, excited type, stupor, withdrawn type; Schizop
295.23	Catatonic Type Schizophrenia, Subchronic State With Acute Exacerbation Catatonic (schizophrenia): agitation, excitation, excited type, stup

295.24	Catatonic Type Schizophrenia, Chronic State With Acute Exacerbation Catatonic (schizophrenia): agitation, excitation, excited type, stupor,
295.25	Catatonic Type Schizophrenia, In Remission Catatonic (schizophrenia): agitation, excitation, excited type, stupor, withdrawn type; Schizophre
295.3	Paranoid Type Schizophrenia Paraphrenic schizophrenia
295.3	Paranoid Type Schizophrenia, Unspecified State Paraphrenic schizophrenia
295.31	Paranoid Type Schizophrenia, Subchronic State Paraphrenic schizophrenia
295.32	Paranoid Type Schizophrenia, Chronic State Paraphrenic schizophrenia
295.33	Paranoid Type Schizophrenia, Subchronic State With Acute Exacerbation Paraphrenic schizophrenia
295.34	Paranoid Type Schizophrenia, Chronic State With Acute Exacerbation Paraphrenic schizophrenia
295.35	Paranoid Type Schizophrenia, In Remission Paraphrenic schizophrenia
295.4	Acute Schizophrenic Episode, Unspecified State Oneirophrenia; Schizophreniform: attack, disorder, psychosis confusional type
295.5	Latent Schizophrenia Latent schizophrenic reaction; Schizophrenia: borderline, incipient, prepsychotic, prodromal, pseudoneurotic, pseudopsyc
295.5	Latent Schizophrenia, Unspecified State Latent schizophrenic reaction; Schizophrenia: borderline, incipient, prepsychotic, prodromal, pseudon
295.51	Latent Schizophrenia, Subchronic State Latent schizophrenic reaction; Schizophrenia: borderline, incipient, prepsychotic, prodromal, pseudon
295.52	Latent Schizophrenia, Chronic State Latent schizophrenic reaction; Schizophrenia: borderline, incipient, prepsychotic, prodromal, pseudoneur
295.53	Latent Schizophrenia, Subchronic State With Acute Exacerbation Latent schizophrenic reaction; Schizophrenia: borderline, incipient, prepsych
295.54	Latent Schizophrenia, Chronic State With Acute Exacerbation Latent schizophrenic reaction; Schizophrenia: borderline, incipient, prepsychoti
295.55	Latent Schizophrenia, In Remission Latent schizophrenic reaction; Schizophrenia: borderline, incipient, prepsychotic, prodromal, pseudoneur
295.6	Residual Schizophrenia Chronic undifferentiated schizophrenia; Restzustand (schizophrenic); Schizophrenic residual state
295.6	Residual Schizophrenia, Unspecified State Chronic undifferentiated schizophrenia; Restzustand (schizophrenic); Schizophrenic residual state
295.61	Residual Schizophrenia, Subchronic State Chronic undifferentiated schizophrenia; Restzustand (schizophrenic); Schizophrenic residual state

295.62	Residual Schizophrenia, Chronic State Chronic undifferentiated schizophrenia; Restzustand (schizophrenic); Schizophrenic residual state
295.63	Residual Schizophrenia, Subchronic State With Acute Exacerbation Chronic undifferentiated schizophrenia; Restzustand (schizophrenic); Schiz
295.64	Residual Schizophrenia, Chronic State With Acute Exacerbation Chronic undifferentiated schizophrenia; Restzustand (schizophrenic); Schizoph
295.65	Residual Schizophrenia, In Remission Chronic undifferentiated schizophrenia; Restzustand (schizophrenic); Schizophrenic residual state
295.7	Schizo- affective type schizophrenia
295.71	Schizo- affective disorder, subchronic
295.72	Schizo- affective disorder, chronic
295.73	Schizo- affective disorder, subchronic with acute exacerbation
295.74	Schizo- affective disorder, chronic with acute exacerbation
295.75	Schizo- affective disorder, in remission
295.8	Other Specified Types Of Schizophrenia Acute (undifferentiated) schizophrenia; Atypical schizophrenia; Cenesthopathic schizophrenia
295.8	Other Specified Types Of Schizophrenia, Unspecified State Acute (undifferentiated) schizophrenia; Atypical schizophrenia; Cenesthopathic schi
295.81	Other Specified Types Of Schizophrenia, Subchronic State Acute (undifferentiated) schizophrenia; Atypical schizophrenia; Cenesthopathic schi
295.82	Other Specified Types Of Schizophrenia, Chronic State Acute (undifferentiated) schizophrenia; Atypical schizophrenia; Cenesthopathic schizop
295.83	Other Specified Types Of Schizophrenia, Subchronic State With Acute Exacerbation Acute (undifferentiated) schizophrenia; Atypical schizophre
295.84	Other Specified Types Of Schizophrenia, Chronic State With Acute Exacerbation Acute (undifferentiated) schizophrenia; Atypical schizophrenia
295.85	Other Specified Types Of Schizophrenia, In Remission Acute (undifferentiated) schizophrenia; Atypical schizophrenia; Cenesthopathic schizop
295.9	Unspecified Schizophrenia Schizophrenia: NOS, mixed NOS, undifferentiated NOS; Schizophrenic reaction NOS; Schizophreniform psychosis NOS
295.9	Unspecified Type Schizophrenia, Unspecified State Schizophrenia: NOS, mixed NOS, undifferentiated NOS; Schizophrenic reaction NOS; Schizoph
295.91	Unspecified Type Schizophrenia, Subchronic State Schizophrenia: NOS, mixed NOS, undifferentiated NOS; Schizophrenic reaction NOS; Schizophren
295.92	Unspecified Type Schizophrenia, Chronic State Schizophrenia: NOS, mixed NOS, undifferentiated NOS; Schizophrenic reaction NOS; Schizophrenifo

295.93	Unspecified Type Schizophrenia, Subchronic State With Acute Exacerbation Schizophrenia: NOS, mixed NOS, undifferentiated NOS; Schizophrenic r
295.94	Unspecified Type Schizophrenia, Chronic State With Acute Exacerbation Schizophrenia: NOS, mixed NOS, undifferentiated NOS; Schizophrenic reac
295.95	Unspecified Type Schizophrenia, In Remission Schizophrenia: NOS, mixed NOS, undifferentiated NOS; Schizophrenic reaction NOS; Schizophrenifo
296	Affective Psychoses
296	Manic Disorder, Single Episode {Hypomania (mild) NOS} {Hypomanic psychosis} {Mania (monopolar) NOS} {Manic
296.1	Manic Affective Disorder, Recurrent Episode, Unspecified Degree Any condition classifiable to 296.0, stated to be recurrent
296.2	Major Depressive Disorder, Single Episode {Depressive psychosis} {Endogenous depression} {Involutional melancholia} {Manic
296.24	Major Depressive Affective Disorder, Single Episode, Severe Degree, Specified As With Psychotic Behavior {Depressive psychosis} {Endogenous
296.26	Major Depressive Affective Disorder, Single Episode, In Full Remission {Depressive psychosis} {Endogenous depression} {Involutional melanch
296.3	Major Depressive Affective Disorder, Recurrent Episode, Unspecified Degree Any condition classifiable to 296.2, stated to be recurrent
296.34	Major Depressive Affective Disorder, Recurrent Episode, Severe Degree, Specified As With Psychotic Behavior Any condition classifiable to 29
296.35	Major Depressive Affective Disorder, Recurrent Episode, In Partial Or Unspecified Remission Any condition classifiable to 296.2, stated to
296.36	Major Depressive Affective Disorder, Recurrent Episode, In Full Remission Any condition classifiable to 296.2, stated to be recurrent
296.4	Bipolar Affective Disorder, Manic Bipolar disorder, now manic; Manic
296.4	Bipolar Affective Disorder, Manic, Unspecified Degree Bipolar disorder, now manic; Manic
296.44	Bipolar Affective Disorder, Manic, Severe Degree,Specified As With Psychotic Behavior Bipolar disorder, now manic; Manic
296.46	Bipolar Affective Disorder, Manic, In Full Remission Bipolar disorder, now manic Manic
296.5	Bipolar Affective Disorder, Depressed Bipolar disorder, now depressed; Manic
296.51	Bipolar Affective Disorder, Depressed, Mild Degree Bipolar disorder, now depressed; Manic

296.52	Bipolar Affective Disorder, Depressed, Moderate Degree Bipolar disorder, now depressed; Manic
296.54	Bipolar Affective Disorder, Depressed, Severe Degree, Specified As With Psychotic Behavior Bipolar disorder, now depressed; Manic
296.56	Bipolar Affective Disorder, Depressed, In Remission Bipolar disorder, now depressed; Manic
296.6	Bipolar Affective Disorder, Mixed Manic
296.6	Bipolar Affective Disorder, Mixed, Unspecified Degree Manic
296.64	Bipolar Affective Disorder, Mixed, Severe Degree, Specified As With Psychotic Behavior Manic
296.65	Bipolar Affective Disorder, Mixed, In Partial Or Unspecified Remission Manic
296.66	Bipolar Affective Disorder, Mixed, In Full Remission Manic
296.7	Bipolar Affective Disorder, Unspecified Atypical bipolar affective disorder NOS; Manic
296.8	Manic
296.82	Atypical Depressive Disorder
296.89	Other Manic
296.9	Unspecified Affective Psychosis Affective psychosis NOS; Melancholia NOS
297	Paranoid States
297.8	Other Specified Paranoid States Paranoia querulans; Sensitiver Beziehungswahn
297.9	Unspecified Paranoid State Paranoid: disorder NOS, psychosis NOS, reaction NOS, state NOS
298	Other Nonorganic Psychoses
298.1	Excitative Type Psychosis Acute hysterical psychosis; Psychogenic excitation; Reactive excitation
298.3	Acute Paranoid Reaction Acute psychogenic paranoid psychosis; Bouffee delirante
298.4	Psychogenic Paranoid Psychosis Protracted reactive paranoid psychosis
298.8	Other And Unspecified Reactive Psychosis Brief reactive psychosis NOS; Hysterical psychosis; Psychogenic psychosis NOS; Psychogenic stupor
298.8	Other And Unspecified Reactive Psychosis Brief reactive psychosis NOS; Hysterical psychosis; Psychogenic psychosis NOS; Psychogenic stupor
298.9	Unspecified Psychosis Atypical psychosis; Psychosis NOS
299.1	Disintegrative Psychosis Heller's syndrome
299.1	Disintegrative Psychosis, Current Or Active State Heller's syndrome
299.11	Disintegrative Psychosis, Residual State Heller's syndrome

299.81	Other Specified Early Childhood Psychoses, Residual State Atypical childhood psychosis; Borderline psychosis of childhood
299.9	Unspecified Childhood Psychosis Child psychosis NOS; Schizophrenia, childhood type NOS; Schizophrenic syndrome of childhood NOS
300	Anxiety State, Unspecified Anxiety: neurosis, reaction, state (neurotic); Atypical anxiety disorder
300.01	Panic Disorder Panic: attack, state
300.02	Generalized Anxiety Disorder
300.09	Other Anxiety States
300.2	Phobic Disorders
300.21	Agoraphobia With Panic Attacks Fear of: {Open spaces} {Streets} {Travel} with panic attacks
300.4	Neurotic Depression Anxiety depression; Depression with anxiety; Depressive reaction; Dysthymic disorder; Neurotic depressive state; Reactiv
301	PARANOID PERSONALITY DISORDER
301.2	Schizoid Personality Disorder, Unspecified
301.22	Schizotypal Personality Disorder
301.5	Histrionic Personality Disorder, Unspecified Hysterical personality NOS
301.6	Dependent Personality Disorder Asthenic personality; Inadequate personality; Passive personality
301.7	Antisocial Personality Disorder Amoral personality; Asocial personality; Dyssocial personality; Personality disorder with predominantly soci
301.81	Narcissistic Personality
301.82	Avoidant Personality Dis
301.83	Borderline Personality
301.9	Unspecified Personality Disorder Pathological personality NOS; Personality disorder NOS; Psychopathic: constitutional state, personality (dis
303	Alcohol Dependence Syndrome
303	Acute Alcoholic Intoxication In Alcoholism, Unspecified Drinking Behavior Acute drunkenness in alcoholism
305	Nondependent DRUG ABUSE
305	Alcohol Abuse, Unspecified Drinking Behavior Drunkenness NOS; Excessive drinking of alcohol NOS; "Hangover" (alcohol); Inebriety NOS
305.01	Alcohol Abuse, Continuous Drinking Behavior Drunkenness NOS; Excessive drinking of alcohol NOS; "Hangover" (alcohol); Inebriety NOS

305.02	Alcohol Abuse, Episodic Drinking Behavior Drunkenness NOS; Excessive drinking of alcohol NOS; "Hangover" (alcohol); Inebriety NOS
305.03	Alcohol Abuse, In Remission Drunkenness NOS; Excessive drinking of alcohol NOS; "Hangover" (alcohol); Inebriety NOS
305.2	Cannabis Abuse, Unspecified Use
305.21	Cannabis Abuse, Continuous Use
305.22	Cannabis Abuse, Episodic Use
305.23	Cannabis Abuse, In Remission
305.3	Hallucinogen Abuse Acute intoxication from hallucinogens ["bad trips"]; LSD reaction
305.31	Hallucinogen Abuse, Continuous Use Acute intoxication from hallucinogens ["bad trips"]; LSD reaction
305.32	Hallucinogen Abuse, Episodic Use Acute intoxication from hallucinogens ["bad trips"]; LSD reaction
305.33	Hallucinogen Abuse, In Remission Acute intoxication from hallucinogens ["bad trips"]; LSD reaction
305.4	Barbiturate And Similarly Acting Sedative Or Hypnotic Abuse
305.41	Barbiturate And Similarly Acting Sedative Or Hypnotic Abuse, Continuous Use
305.42	Barbiturate And Similarly Acting Sedative Or Hypnotic Abuse, Episodic Use
305.43	Barbiturate And Similarly Acting Sedative Or Hypnotic Abuse, In Remission
305.5	Opioid Abuse, Unspecified Use
305.51	Opioid Abuse, Continuous Use
305.52	Opioid Abuse, Episodic Use
305.53	Opioid Abuse, In Remission
305.6	Cocaine Abuse, Unspecified Use
305.61	Cocaine Abuse, Continuous Use
305.62	Cocaine Abuse, Episodic Use
305.63	Cocaine Abuse, In Remission
305.7	Amphetamine Or Related Acting Sympathomimetic Abuse, Unspecified Use
305.71	Amphetamine Or Related Acting Sympathomimetic Abuse, Continuous Use
305.72	Amphetamine Or Related Acting Sympathomimetic Abuse, Episodic Use
305.73	Amphetamine Or Related Acting Sympathomimetic Abuse, In Remission
305.8	Antidepressant Type Abuse
305.81	Antidepressant Type Abuse, Continuous Use

305.82	Antidepressant Type Abuse, Episodic Use
305.83	Antidepressant Type Abuse, In Remission
305.9	Other, Mixed, Or Unspecified Drug Abuse "Laxative habit"; Misuse of drugs NOS; Nonprescribed use of drugs or patent medicinals
305.9	Other, Mixed, Or Unspecified Drug Abuse, Unspecified Use "Laxative habit"; Misuse of drugs NOS; Nonprescribed use of drugs or patent medicin
305.91	Other, Mixed, Or Unspecified Drug Abuse, Continuous Use "Laxative habit"; Misuse of drugs NOS; Nonprescribed use of drugs or patent medicin
305.92	Other, Mixed, Or Unspecified Drug Abuse, Episodic Use "Laxative habit"; Misuse of drugs NOS; Nonprescribed use of drugs or patent medicinals
305.93	Other, Mixed, Or Unspecified Drug Abuse, In Remission "Laxative habit"; Misuse of drugs NOS; Nonprescribed use of drugs or patent medicinals
308	Predominant Disturbance Of Emotions {Anxiety} {Emotional crisis} {Panic state} as acute reaction to exceptional [gross] stress
309	Adjustment Reaction With Brief Depressive Reaction Adjustment disorder with depressed mood; Grief reaction
309	Adjustment Reaction With Brief Depressive Reaction Adjustment disorder with depressed mood; Grief reaction
309.1	Adjustment Reaction With Prolonged Depressive Reaction
309.3	Adjustment Reaction With Predominant Disturbance Of Conduct {Conduct disturbance} {Destructiveness} as adjustment reaction
309.4	Adjustment Reaction With Mixed Disturbance Of Emotions And Conduct
310.1	Organic Personality Syndrome Cognitive or personality change of other type, of nonpsychotic severity; Mild memory disturbance; Organic psych
311	Depressive Disorder, NEC Depressive disorder NOS; Depressive state NOS; Depression NOS
312	Undersocialized Conduct Disorder, Aggressive Type Aggressive outburst; Anger reaction; Unsocialized aggressive disorder
312.1	Undersocialized Conduct Disorder, Unaggressive Type Childhood truancy, unsocialized; Solitary stealing; Tantrums
312.4	Mixed Disturbance Of Conduct And Emotions Neurotic delinquency
312.8	Other Specified disturbances of Conduct, NEC
312.9	Unspecified Disturbance Of Conduct Delinquency (juvenile)
314	Attention Deficit Disorder Adult, Child
314	Attention Deficit Disorder Of Childhood Without Mention Of Hyperactivity Predominantly inattentive type

314.01	Attention Deficit Disorder Of Childhood With Hyperactivity Combined type; Overactivity NOS; predominantly hyperactive/impulsive type; Simpl
314.9	Unspecified Hyperkinetic Syndrome Of Childhood Hyperkinetic reaction of childhood or adolescence NOS; Hyperkinetic syndrome NOS
317	MENTAL RETARDATION MILD IQ 50
318	Other Specified Mental Retardation
318.1	Severe Mental Retardation IQ 20
318.2	Profound Mental Retardation IQ under 20; Profoundmental subnormality
319.9	MENTAL RETARDATION UNSPECIFIED
357.5	Alcoholic Polyneuropathy
425.5	Alcoholic Cardiomyopathy
535.3	Alcoholic Gastritis
571	Alcoholic Fatty Liver
571.1	Acute Alcoholic Hepatitis Acute alcoholic liver disease
571.2	Alcoholic Cirrhosis Of Liver Florid cirrhosis; Laennec's cirrhosis (alcoholic)
571.3	Alcoholic Liver Damage, Unspecified
790.3	Excessive Blood Level Of Alcohol Elevated blood
980.9	Toxic Effect Of Unspecified Alcohol
V40.00	BORDERLINE INTELLECTUAL FUNCTIONING
V69.1	Inappropriate diet and eating habits
V71.02	Observation Of Childhood Or Adolescent Antisocial Behavior Dyssocial behavior or gang activity in child or adolescent without manifest psyc
300.09	Other Anxiety States
309.1	Adjustment Reaction With Prolonged Depressive Reaction
294.9	Unspecified Organic Brain Syndrome (chronic) Organic psychosis (chronic)
295.1	Disorganized Type Schizophrenia, Unspecified State Hebephrenia; Hebephrenic type schizophrenia
295.2	Catatonic Type Schizophrenia, Unspecified State Catatonic (schizophrenia): agitation, excitation, excited type, stupor, withdrawn type; Schi
295.3	Paranoid Type Schizophrenia, Unspecified State Paraphrenic schizophrenia
295.4	Acute Schizophrenic Episode, Unspecified State Oneirophrenia; Schizophreniform: attack, disorder, psychosis confusional type
295.6	Residual Schizophrenia, Unspecified State Chronic undifferentiated schizophrenia; Restzustand (schizophrenic); Schizophrenic residual state

295.7	Schizo
295.9	Unspecified Type Schizophrenia, Unspecified State Schizophrenia: NOS, mixed NOS, undifferentiated NOS; Schizophrenic reaction NOS; Schizoph
296.8	Manic
299.81	Other Specified Early Childhood Psychoses, Residual State Atypical childhood psychosis; Borderline psychosis of childhood
309	Adjustment Reaction With Brief Depressive Reaction Adjustment disorder with depressed mood; Grief reaction
314	Attention Deficit Disorder Of Childhood Without Mention Of Hyperactivity Predominantly inattentive type
295.5	Latent Schizophrenia Latent schizophrenic reaction; Schizophrenia: borderline, incipient, prepsychotic, prodromal, pseudoneurotic, pseudopsyc
295.11	Disorganized Type Schizophrenia, Subchronic State Hebephrenia; Hebephrenic type schizophrenia
295.12	Disorganized Type Schizophrenia, Chronic State Hebephrenia; Hebephrenic type schizophrenia
295.13	Disorganized Type Schizophrenia, Subchronic State With Acute Exacerbation Hebephrenia; Hebephrenic type schizophrenia
295.14	Disorganized Type Schizophrenia, Chronic State With Acute Exacerbation Hebephrenia; Hebephrenic type schizophrenia
295.15	Disorganized Type Schizophrenia, In Remission Hebephrenia; Hebephrenic type schizophrenia
295.21	Catatonic Type Schizophrenia, Subchronic State Catatonic (schizophrenia): agitation, excitation, excited type, stupor, withdrawn type; Schi
295.22	Catatonic Type Schizophrenia, Chronic State Catatonic (schizophrenia): agitation, excitation, excited type, stupor, withdrawn type; Schizop
295.23	Catatonic Type Schizophrenia, Subchronic State With Acute Exacerbation Catatonic (schizophrenia): agitation, excitation, excited type, stup
295.24	Catatonic Type Schizophrenia, Chronic State With Acute Exacerbation Catatonic (schizophrenia): agitation, excitation, excited type, stupor,
295.25	Catatonic Type Schizophrenia, In Remission Catatonic (schizophrenia): agitation, excitation, excited type, stupor, withdrawn type; Schizophre
295.31	Paranoid Type Schizophrenia, Subchronic State Paraphrenic schizophrenia
295.32	Paranoid Type Schizophrenia, Chronic State Paraphrenic schizophrenia
295.33	Paranoid Type Schizophrenia, Subchronic State With Acute Exacerbation Paraphrenic schizophrenia

295.34	Paranoid Type Schizophrenia, Chronic State With Acute Exacerbation Paraphrenic schizophrenia
295.35	Paranoid Type Schizophrenia, In Remission Paraphrenic schizophrenia
295.91	Unspecified Type Schizophrenia, Subchronic State Schizophrenia: NOS, mixed NOS, undifferentiated NOS; Schizophrenic reaction NOS; Schizophren
295.92	Unspecified Type Schizophrenia, Chronic State Schizophrenia: NOS, mixed NOS, undifferentiated NOS; Schizophrenic reaction NOS; Schizophrenifo
295.93	Unspecified Type Schizophrenia, Subchronic State With Acute Exacerbation Schizophrenia: NOS, mixed NOS, undifferentiated NOS; Schizophrenic r
295.94	Unspecified Type Schizophrenia, Chronic State With Acute Exacerbation Schizophrenia: NOS, mixed NOS, undifferentiated NOS; Schizophrenic reac
295.95	Unspecified Type Schizophrenia, In Remission Schizophrenia: NOS, mixed NOS, undifferentiated NOS; Schizophrenic reaction NOS; Schizophrenifo
295	Simple Type Schizophrenia Schizophrenia simplex
296.9	Unspecified Affective Psychosis Affective psychosis NOS; Melancholia NOS
299	Infantile Autism Childhood autism; Infantile psychosis; Kanner's syndrome
298.9	Unspecified Psychosis Atypical psychosis; Psychosis NOS
295.3	Paranoid Type Schizophrenia Paraphrenic schizophrenia
295.1	Disorganized Type Schizophrenia Hebephrenia; Hebephrenic type schizophrenia
295.2	Catatonic Type Schizophrenia Catatonic (schizophrenia): agitation, excitation, excited type, stupor, withdrawn type; Schizophrenic: cataleps
295.64	Residual Schizophrenia, Chronic State With Acute Exacerbation Chronic undifferentiated schizophrenia; Restzustand (schizophrenic); Schizoph
295.63	Residual Schizophrenia, Subchronic State With Acute Exacerbation Chronic undifferentiated schizophrenia; Restzustand (schizophrenic); Schiz
295.65	Residual Schizophrenia, In Remission Chronic undifferentiated schizophrenia; Restzustand (schizophrenic); Schizophrenic residual state
295.6	Residual Schizophrenia Chronic undifferentiated schizophrenia; Restzustand (schizophrenic); Schizophrenic residual state
295.62	Residual Schizophrenia, Chronic State Chronic undifferentiated schizophrenia; Restzustand (schizophrenic); Schizophrenic residual state
295.61	Residual Schizophrenia, Subchronic State Chronic undifferentiated schizophrenia; Restzustand (schizophrenic); Schizophrenic residual state
297.8	Other Specified Paranoid States Paranoia querulans; Sensitiver Beziehungswahn
297.9	Unspecified Paranoid State Paranoid: disorder NOS, psychosis NOS, reaction NOS, state NOS

298.1	Excitative Type Psychosis Acute hysterical psychosis; Psychogenic excitation; Reactive excitation
298.8	Other And Unspecified Reactive Psychosis Brief reactive psychosis NOS; Hysterical psychosis; Psychogenic psychosis NOS; Psychogenic stupor
298	Other Nonorganic Psychoses
295.5	Latent Schizophrenia, Unspecified State Latent schizophrenic reaction; Schizophrenia: borderline, incipient, prepsychotic, prodromal, pseudon
295.51	Latent Schizophrenia, Subchronic State Latent schizophrenic reaction; Schizophrenia: borderline, incipient, prepsychotic, prodromal, pseudon
295.52	Latent Schizophrenia, Chronic State Latent schizophrenic reaction; Schizophrenia: borderline, incipient, prepsychotic, prodromal, pseudoneur
295.53	Latent Schizophrenia, Subchronic State With Acute Exacerbation Latent schizophrenic reaction; Schizophrenia: borderline, incipient, prepsych
295.54	Latent Schizophrenia, Chronic State With Acute Exacerbation Latent schizophrenic reaction; Schizophrenia: borderline, incipient, prepsychoti
295.55	Latent Schizophrenia, In Remission Latent schizophrenic reaction; Schizophrenia: borderline, incipient, prepsychotic, prodromal, pseudoneur
295.71	Schizo- affective disorder, subchronic
295.72	Schizo- affective disorder, chronic
295.73	Schizo- affective disorder, subchronic with acute exacerbation
295.74	Schizo- affective disorder, chronic with acute exacerbation
295.75	Schizo- affective disorder, in remission
295.84	Other Specified Types Of Schizophrenia, Chronic State With Acute Exacerbation Acute (undifferentiated) schizophrenia; Atypical schizophrenia
295.85	Other Specified Types Of Schizophrenia, In Remission Acute (undifferentiated) schizophrenia; Atypical schizophrenia; Cenesthopathic schizop
295.83	Other Specified Types Of Schizophrenia, Subchronic State With Acute Exacerbation Acute (undifferentiated) schizophrenia; Atypical schizophre
295.82	Other Specified Types Of Schizophrenia, Chronic State Acute (undifferentiated) schizophrenia; Atypical schizophrenia; Cenesthopathic schizop
295.8	Other Specified Types Of Schizophrenia, Unspecified State Acute (undifferentiated) schizophrenia; Atypical schizophrenia; Cenesthopathic schi
295.8	Other Specified Types Of Schizophrenia Acute (undifferentiated) schizophrenia; Atypical schizophrenia; Cenesthopathic schizophrenia
295.81	Other Specified Types Of Schizophrenia, Subchronic State Acute (undifferentiated) schizophrenia; Atypical schizophrenia; Cenesthopathic schi

295.9	Unspecified Schizophrenia Schizophrenia: NOS, mixed NOS, undifferentiated NOS; Schizophrenic reaction NOS; Schizophreniform psychosis NOS
295	Schizophrenic Disorders
299.1	Disintegrative Psychosis Heller's syndrome
299.1	Disintegrative Psychosis, Current Or Active State Heller's syndrome
299.11	Disintegrative Psychosis, Residual State Heller's syndrome
299.9	Unspecified Childhood Psychosis Child psychosis NOS; Schizophrenia, childhood type NOS; Schizophrenic syndrome of childhood NOS
295.04	Simple Type Schizophrenia, Chronic State With Acute Exacerbation Schizophrenia simplex
295.03	Simple Type Schizophrenia, Subchronic State With Acute Exacerbation Schizophrenia simplex
295.02	Simple Type Schizophrenia, Chronic State Schizophrenia simplex
295.01	Simple Type Schizophrenia, Subchronic State Schizophrenia simplex
295.05	Simple Type Schizophrenia, In Remission Schizophrenia simplex

Supplementary Table 3. Demographic characterizations of the patients stratified by first stimulant purchased. P-value was <0.001 in all parameters. ADHD – attention deficit disorder. SES – Socioeconomic status. SEX-Male/Female. SMD – standardized mean differences.

	Dexmethylphenidate	Dextroamphetamine	Lisdexamfetamine	Methylphenidate	SMD
	(n=806; 1.8%)	(n=4,699; 10.7%)	(n=654; 1.5%)	(n=37,666; 85.9%)	
	n (%)	n (%)	n (%)	n (%)	
Sex = M (%)	523 (64.9)	2761 (58.8)	406 (62.1)	22853 (60.7)	0.07
Age at ADHD diagnosis (median [IQR])	9.95 [7.83, 12.86]	9.99 [7.88, 13.33]	11.68 [8.95, 14.42]	9.44 [7.57, 12.78]	0.23
Age at first ADHD medication (median [IQR])	10.90 [8.58, 13.63]	11.33 [8.87, 14.34]	12.63 [10.13, 15.00]	10.24 [8.17, 13.47]	0.27

Time from Diagnosis to Medication (years) (median [IQR])		0.10 [0.01, 0.85]	0.12 [0.01, 0.97]	0.06 [0.01, 0.57]	0.05 [0.00, 0.55]	0.10
Follow up (years) (median [IQR])*		3.00 [2.00, 5.00]	2.00 [2.00, 4.00]	3.00 [2.00, 4.00]	4.00 [2.00, 6.00]	0.39
Time without medications (gap years) (mean (SD))		0.27 (0.75)	0.14 (0.50)	0.15 (0.57)	0.41 (0.97)	0.21
Index Year (median [IQR])		2019 [2017,2021]	2021 [2020,2022]	2021 [2019,2022]	2018 [2016,2020]	0.67
Parental Psychiatric History (%)		0.69 (0.46)	0.68 (0.47)	0.67 (0.47)	0.69 (0.46)	0.03
Age at first at ADHD medication	6-12	485 (60.2)	2637 (56.1)	282 (43.1)	24199 (64.2)	0.232
	13-16	231 (28.7)	1485 (31.6)	282 (43.1)	9944 (26.4)	
	17-18	90 (11.2)	577 (12.3)	90 (13.8)	3523 (9.4)	
SES	High	168 (20.8)	1057 (22.5)	178 (27.2)	8312 (22.1)	0.10
	Medium	290 (36)	1693 (36.0)	206 (31.5)	12919 (34.3)	
	Low	269 (33.4)	1496 (31.8)	194 (29.7)	12876 (34.2)	
	Very Low	79 (9.8)	453 (9.6)	76 (11.6)	3559 (9.4)	
Persistent User = Yes (%)		390 (48.4)	1657 (35.3)	340 (52.0)	16396 (43.5)	0.19

Supplemental table 4. Multivariate Logistic regression for Sensitivity analysis using a generalized linear model (GLM) with exact matching on follow-up time

		OR	95% CI	P-Value
Sex	Males vs. females	1.46	1.39-1.53	<0.001
SES	High	0.74	0.68-0.82	<0.001
	Medium	0.68	0.62-0.74	<0.001
	Low	0.76	0.70-0.84	<0.001
	Very Low	1 (ref)		
Age Group	6-12	1.65	1.50-1.83	<0.001
	13-16	1.25	1.13-1.39	<0.001
	17-18	1 (ref)		
Index year (per year)		1.13	1.10-1.16	<0.001
Time from ADHD diagnosis to medication (per 1 year)		0.97	0.95-0.99	<0.001
First ADHD Medication	Lisdexamfetamine	1.79	1.47-2.19	<0.001
	Dextroamphetamine	0.89	0.82-0.97	0.006
	Dexmethylphenidate	1.25	1.05-1.50	0.013
	Methylphenidate	1 (ref)		

ADHD – attention deficit disorder; SES – Socioeconomic status

Supplemental table 5. Demographic characterizations of patients included in the sensitivity analyses with exact matching on follow-up time. ADHD – attention deficit disorder. SES – Socioeconomic status. Sex – Male/Female. SMD – standardized mean differences

		**Non-Persistent (n=13,634)**	**Persistent (n=13,634)**	**SMD**
		n (%)	n (%)	
Sex = M (%)		7601 (51.8)	9051 (66.4)	0.22
Time from Diagnosis to Medication (years) (median [IQR])		0.07 [0,0.64]	0.04 [0,0.44]	0.20
Follow up (years) (median [IQR])		4 [3,6]	4 [3,6]	<.0001
Index Year		2018 [2016,2020]	2019 [2017,2021]	.013
Age at first at ADHD medication	6-12	8498 (62.3)	9711 (71.2)	.020
	13-16	3943 (28.9)	3194 (23.4)	
	17-18	1193 (8.8)	729 (5.3)	
SES	High	3234 (23.7)	3036 (22.3)	0.13
	Medium	4898 (35.9)	4396 (32.2)	
	Low	4430 (32.5)	4664 (34.2)	
	Very Low	1072 (7.9)	1538 (11.3)	
First ADHD Medication (%)	Dexmethylphenidate	229 (1.7)	292 (2.1)	.008
	Dextroamphetamine	1490 (10.9)	1490 (10.9)	
	Lisdexamfetamine	158 (1.2)	286 (2.1)	
	Methylphenidate	11706 (85.9)	11566 (84.8)	
Number of medication groups (median [IQR])		2 [1,2]	2 [1,2]	.001

Supplemental Table 6. Demographic characterizations of the patients included in the Sub-Analysis of years 2009-2014 when only methylphenidate was available to treat ADHD in Israel.

		**Non-Persistent (n=15,633)**	**Persistent (n=14,086)**	**SMD**	**P-Value**
Sex = M (%)		9,203 (58.9)	9,246 (65.6)	0.14	<0.001
SES (%)	High	3,484 (22.3)	3,636 (25.8)	0.10	<0.001
	Medium	5,565 (35.6)	4,928 (35)		
	Low	5,415 (34.6)	4,343 (30.8)		
	Very Low	1,169 (7.5)	1,179 (8.4)		
First Diagnosis age (years) (mean (SD))		11.56 (3.33)	9.75 (3.11)	0.56	<0.001
Age at medication start (years) (mean (SD))		11.74 (3.23)	9.87 (3.03)	0.60	<0.001
Age Group at medication start (%)	06-12	8,608 (53.1)	10,692 (75.9)	0.49	<0.001
	13-16	5,487 (35.1)	2,729 (19.4)		
	17-18	1,838 (11.8)	665 (4.7)		

Supplemental Figures

Supplemental Figure 1. Monthly distribution of ADHD medication purchases across the study period, demonstrating lower purchase rates during extended school breaks (April, July and August).

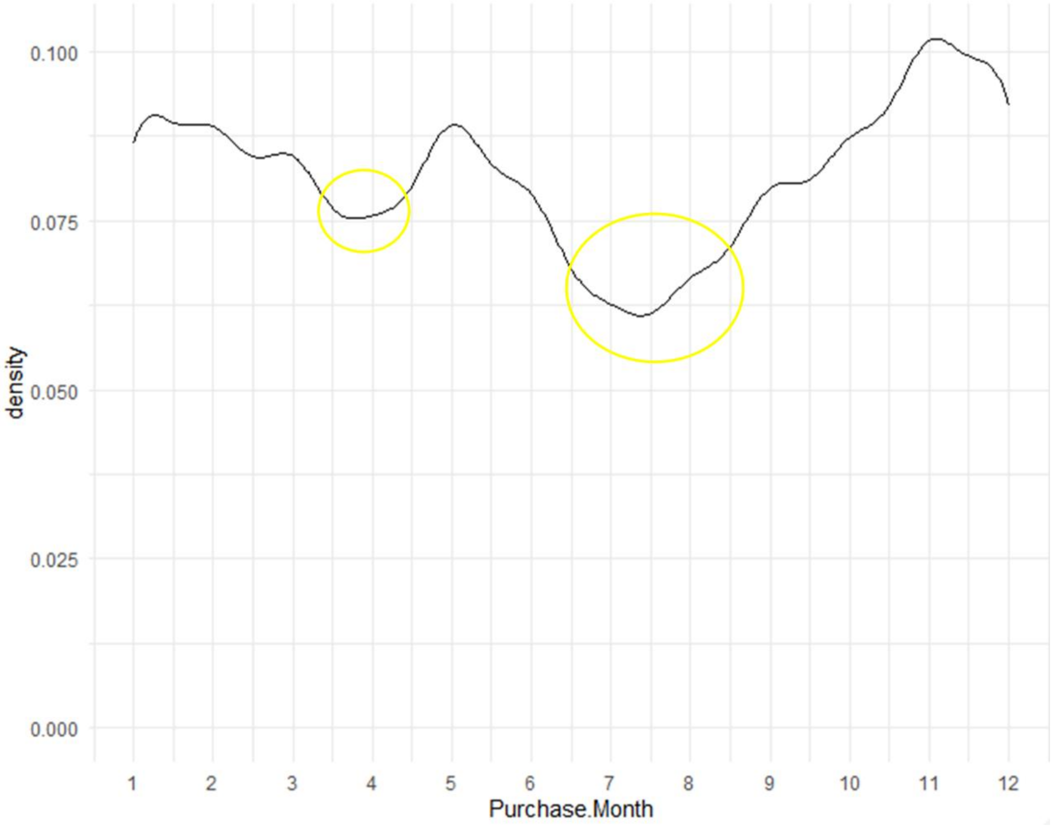

Supplemental Figure 2. Proportion of patients who used only one medication group throughout the study period, stratified by age group and medication class.

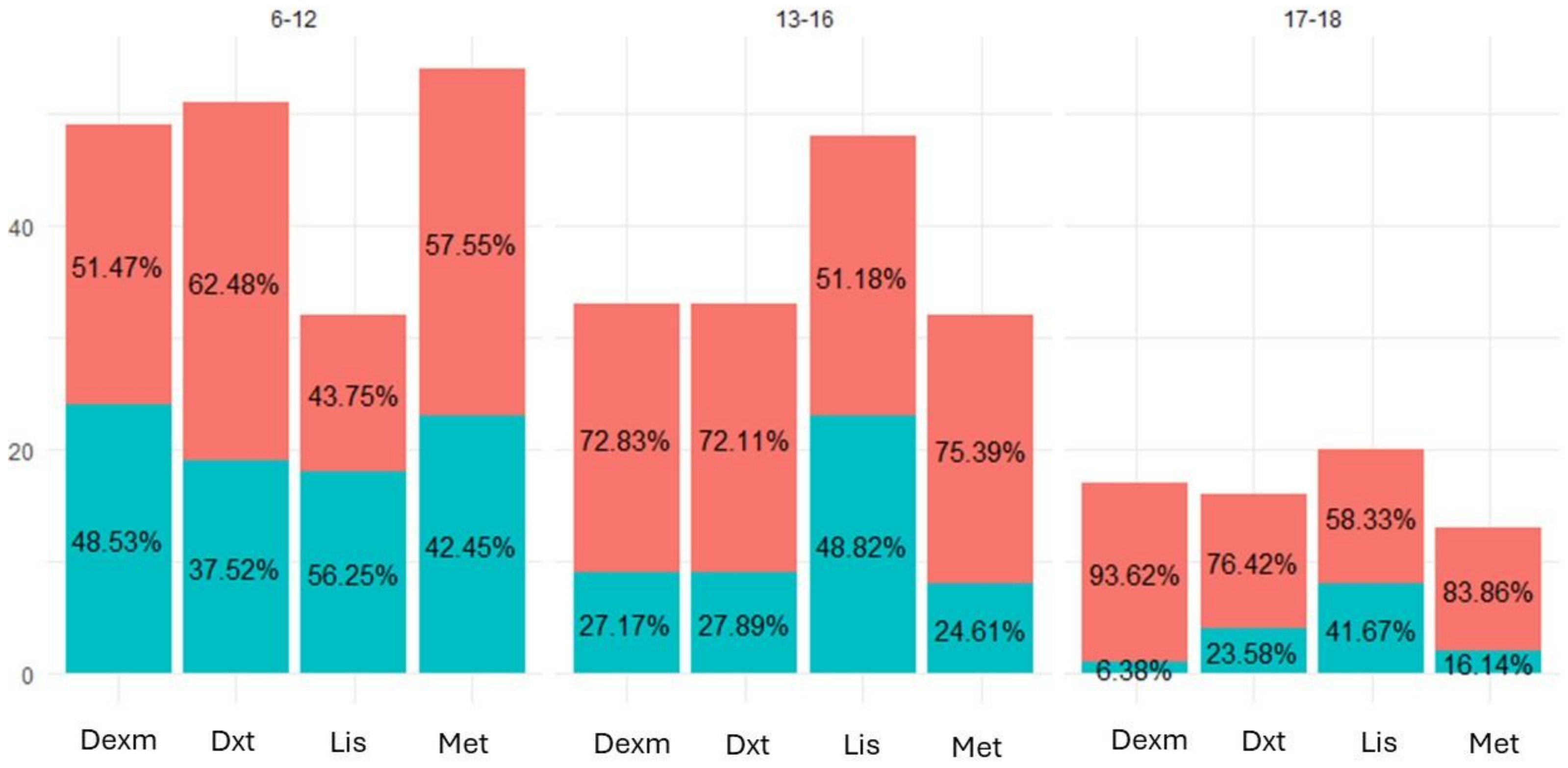

Each bar represents the percentage within the population, with the inner distribution indicating the rate of persistent (green) and inpersistent (red) medication use within each group. The x-axis represents the type of stimulant purchased stratified by age groups, while the y-axis represents the percentage of patients within each group. Among the older age groups, the Lisdexamfetamine group appears to exhibit the highest rate of persistent medication use. The Dextroamphetamine group shows the highest proportion of users who remained on a single medication group without switching among children aged 6-12.

Dexm - dexmethylphenidate; Dxt - dextroamphetamine; Lis - lisdexamphetamine; Met – methylphenidate.

Supplemental Figure 3. The association between the first medication purchased and the medication persistently used by the patient.

		Persistent use				Non-Persistent
		Dxt	Met	Dexm	Lis	
First Medication	Dxt	28%	4%	1%	3%	64%
	Met	6%	34%	2%	2%	56%
	Dexm	8%	7%	29%	4%	51%
	Lis	6%	4%	2%	41%	48%

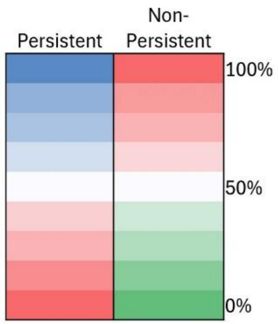

The chi-square test indicates a statistically significant association ($p < 0.0001$). The strength of this association is supported by Cramér's V (0.4), indicating a relatively strong relationship, persistent with the contingency coefficient result (0.59). Data covers the years 2015-2023. Dexm - dexmethylphenidate; Dxt - dextroamphetamine; Lis - lisdexamphetamine; Met – methylphenidate.

Supplemental Figure 4. Heatmaps showing consistency of medication use by first medication type and age group

Age Group 6-12

		Persistent use				Non-Persistent
		Dxt	Met	Dexm	Lis	
First Medication	Dxt	791	170	39	76	1561
	Met	1691	9937	549	637	11385
	Dexm	45	41	183	22	194
	Lis	21	20	10	113	118

Age Group 6-12

		Persistent use				Non-Persistent
		Dxt	Met	Dexm	Lis	
First Medication	Dxt	30%	6%	1%	3%	59%
	Met	7%	41%	2%	3%	47%
	Dexm	9%	8%	38%	5%	40%
	Lis	7%	7%	4%	40%	42%

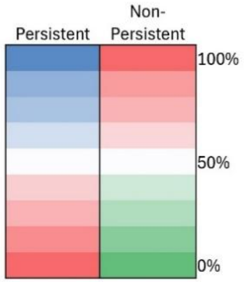

Age Group 13-16

		Persistent use				Non-Persistent
		Dxt	Met	Dexm	Lis	
First Medication	Dxt	380	27	4	40	1034
	Met	413	2376	63	163	6929
	Dexm	19	13	43	9	147
	Lis	13	5	2	118	144

Age Group 13-16

		Persistent use				Non-Persistent
		Dxt	Met	Dexm	Lis	
First Medication	Dxt	26%	2%	0%	3%	70%
	Met	4%	24%	1%	2%	70%
	Dexm	8%	6%	19%	4%	64%
	Lis	5%	2%	1%	42%	51%

Age Group 17-18

		Persistent use				Non-Persistent
		Dxt	Met	Dexm	Lis	
First Medication	Dxt	131	10	1	5	430
	Met	128	569	10	42	2774
	Dexm	2	3	11	2	72
	Lis	2	2	0	37	49

Age Group 17-18

		Persistent use				Non-Persistent
		Dxt	Met	Dexm	Lis	
First Medication	Dxt	23%	2%	0%	1%	75%
	Met	4%	16%	0%	1%	79%
	Dexm	2%	3%	12%	2%	80%
	Lis	2%	2%	0%	41%	54%

. Higher within-group consistency is observed for Methylphenidate in younger age groups and for Lisdexamfetamine in older groups. Non persistent rates decrease with age for some medications, notably Lisdexamfetamine. Overall, Lisdexamfetamine shows increasing consistency and decreasing failure rates across age groups, in contrast to Methylphenidate, which dominates in younger ages but declines in effectiveness with age. Full percentages and counts are presented for each combination. Dexm - dexmethylphenidate; Dxt - dextroamphetamine; Lis - lisdexamphetamine; Met – methylphenidate.

Supplemental Figure 5a-d. Percent of persistent users by age and sex, with 95% CI

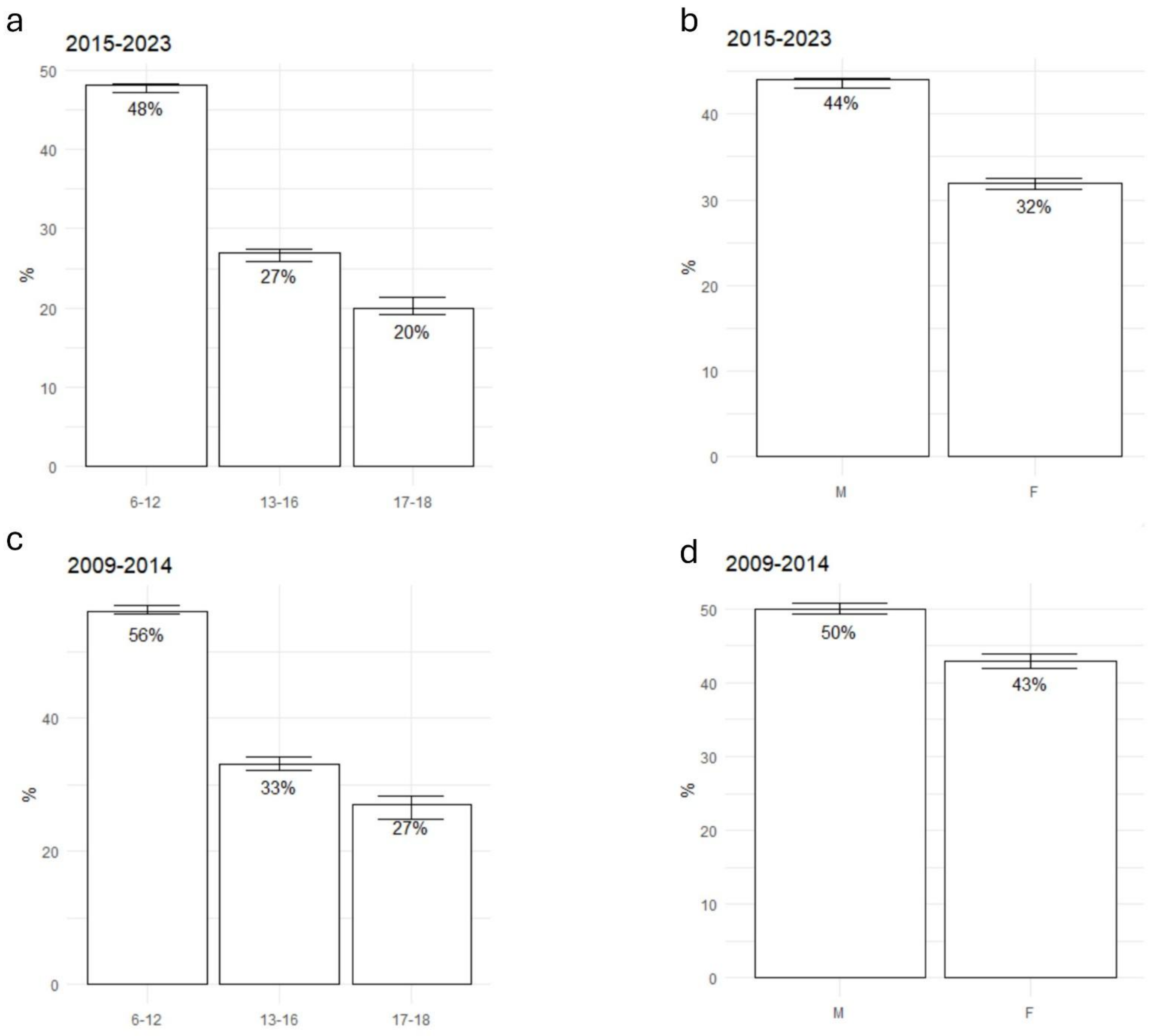

.

The figure displays the first medication use by age group (6–12, 13–16, and 17–18 years). This figure illustrates a higher rate of persistent medication use in the youngest age group and in males, as well as a higher proportion of children initiating medication, in both sub analysis (2009-2014) and primary analysis (2015-2023). Between 2009 and 2014, only methylphenidate was available, suggesting that the pattern of use observed over the full study period is not solely driven by the type of medication.
